# Large‐Area Virus Coated Ultrathin Colorimetric Sensors with a Highly Lossy Resonant Promoter for Enhanced Chromaticity

**DOI:** 10.1002/advs.202000978

**Published:** 2020-07-21

**Authors:** Young Jin Yoo, Won‐Geun Kim, Joo Hwan Ko, Yeong Jae Kim, Yujin Lee, Stefan G. Stanciu, Jong‐Min Lee, Seungchul Kim, Jin‐Woo Oh, Young Min Song

**Affiliations:** ^1^ School of Electrical Engineering and Computer Science Gwangju Institute of Science and Technology Gwangju 61005 Republic of Korea; ^2^ Department of Nano Fusion Technology Pusan National University Busan 46241 Republic of Korea; ^3^ Center for Microscopy‐Microanalysis and Information Processing Politehnica University Bucharest Bucharest 060042 Romania; ^4^ Research Center for Energy Convergence and Technology Pusan National University Busan 46241 Republic of Korea; ^5^ Department of Optics and Mechatronics Engineering Pusan National University Busan 46241 Republic of Korea; ^6^ Department of Nano Fusion Technology Pusan National University Busan 46241 Republic of Korea; ^7^ Department of Nanoenergy Engineering Pusan National University Busan 46241 Republic of Korea; ^8^ BK21 PLUS Nanoconvergence Technology Division Pusan National University Busan 46241 Republic of Korea; ^9^ Anti‐Viral Research Center Gwangju Institute of Science and Technology Gwangju 61005 Republic of Korea; ^10^ AI Graduate School Gwangju Institute of Science and Technology Gwangju 61005 Republic of Korea

**Keywords:** bacteriophages, colorimetric sensors, endocrine disrupting chemicals, thin‐film coloration, volatile organic compounds

## Abstract

Acclimatable colors in response to environmental stimuli, which are naturally endowed with some living things, can provide an opportunity for humans to recognize hazardous substances without taking empirical risks. Despite efforts to create artificial responsive colors, realistic applications in everyday life require an immediate/distinct colorimetric realization with wide chromatic selectivity. A dynamically responsive virus (M‐13 phage)‐based changeable coloring strategy is presented with a highly lossy resonant promoter (HLRP). An ultrathin M‐13 phage layer for rapid response to external stimuli displays colorimetric behavior, even in its subtle swelling with strong resonances on HLRP, which is modeled using the complex effective refractive index. Optimal designs of HLRP for several material combinations allow selective chromatic responsivity from the corresponding wide color palette without modification of the dynamic responsive layer. As a practical demonstration, the spatially designed colorimetric indicator, which is insensitive/sensitive to external stimuli, provides an intuitive perception of environmental changes with hidden/revealed patterns. Furthermore, the proposed colorimetric sensor is tested by exposure to various volatile organic chemicals and endocrine disrupting chemicals for versatile detectability, and is fabricated in a wafer‐scale sample for large‐area scalability.

## Graphical abstract

A large‐area virus coated ultrathin colorimetric sensor with a highly lossy resonant promoter (HLRP) is developed. A dynamically responsive virus‐based changeable coloring strategy with HLRP is an efficacious approach to fuse the advantages of strong resonant photonic structures and dynamically responsive materials. An ultrathin virus layer for rapid response to external stimuli displays colorimetric behavior on HLRP.

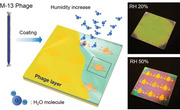

Certain living organisms, such as cephalopods, chameleons, and turkeys, modify their colors to adapt to environmental changes for acclimatization, camouflage, and communication.^[^ ^1^, ^2^ ^]^ These adaptive colors are realized by the change in volume or thickness of cell or protein soft layers in response to environmental stimuli, such as a change in humidity, light, temperature, or concentrations of specific molecules.^[^ ^3^, ^4^ ^]^ Visual self‐adaptation is also required for protection in modern societies having diverse pollutants, as realized, for example, from the fact that weather forecasts include a forecast of air quality.^[^ ^5^ ^]^ Hence, artificially color‐changeable materials, which are environmentally responsive and/or inspired by nature, can be an intuitive monitor beyond the human senses.^[^ ^6^, ^7^, ^8^ ^]^

By recent developments in photonic structures, some structural colors can be reversibly changed in response to external stimuli.^[^ ^9^, ^10^, ^11^, ^12^, ^13^, ^14^, ^15^, ^16^, ^17^, ^18^, ^19^, ^20^, ^21^, ^22^, ^23^, ^24^ ^]^ Based on a lesson from natural photonic structure, specific examples of photonic crystal‐based structural coloring schemes show unprecedented possibilities in colorimetric sensor applications, such as temperature, pH, ion species, solvents, water vapor, humidity, pressure, and biomolecule detection.^[^ ^13^, ^14^, ^15^, ^16^, ^17^, ^18^, ^19^, ^20^, ^21^, ^22^, ^23^, ^24^ ^]^ Since a combination of introduced design methods for colorimetric detection, inspired by photonic structures, depends on highly ordered structures and periodically arranged refractive indices, concomitant co‐assembling of multiple materials and micro/nano building blocks to achieve uniform colors at a large scale constrains their rapid/convenient fabrication.^[^ ^17^, ^18^, ^19^, ^20^ ^]^ Meanwhile, recent approaches utilizing a combination of simple photonic structures with materials that are dynamically responsive to external stimuli, such as proteins and hydrogels, have been successfully demonstrated.^[^ ^23^, ^24^ ^]^ Nevertheless, unoptimized bulky responsive layers and chromaticities that lack wide selectivity make it difficult to guarantee the requirements for realistic applications, such as rapid response time and selective chromatic responsivity.

Herein, we present an efficacious approach to colorimetric sensor design to fuse the advantages of strong resonant photonic structures and dynamically responsive materials. As a reliable dynamic reactant, we employed M‐13 bacteriophages with the capability to generate identical copies of themselves through bacterial host cells infection.^[^ ^25^, ^26^ ^]^ By using a highly lossy resonant promoter (HLRP) as the substrate, the spin‐coated M‐13 virus layer exhibits strong resonance even with ultrathin thickness variations, resulting in colorimetric behavior with enhanced chromaticity. For practical demonstration with selective chromatic responsivity, the colorimetric indicator designed as separate insensitive/sensitive areas reveals hidden patterns at high humidity, allowing intuitive perception of environmental changes. In the design process, we optimize the resonance characteristics according to the complex refractive index of the substrate to maximize the resonance of the coating layer. Furthermore, considering various material combinations, rigorous coupled‐wave analysis (RCWA) is performed to demonstrate a colorimetric palette of wide chromatic selectivity with dynamic variation.^[^ ^27^ ^]^ In addition, using genetically engineered M‐13 phage, we conduct sensing tests on volatile organic chemicals (VOCs) and endocrine disrupting chemicals (EDCs) as an application for detecting diverse substances. For scalability, we also fabricate a large area sample at a wafer scale.

**Figure** **1**a illustrates a schematic of a virus‐coated colorimetric sensor with HLRP. Adopted as a dynamic responsive layer, the M‐13 virus layer causes colorimetric behavior on the HLRP by swelling/deswelling with external changes.^[^ ^28^ ^]^ The virus solution was coated over a highly lossy porous medium on the metal, forming an ultrathin layer by the bundle of the M‐13 phage, which is a bacterial virus composed of single‐stranded DNA encapsulated with various major and minor coat protein (Figure 1b). Such an ultrathin phage layer is facilitated by strong resonance due to interactions at the interface of the HLRP with complex optical constants (Figure 1c).^[^ ^29^, ^30^ ^]^ As a result, the fabricated sample with the hydrophilic phage layer exhibits a distinct color change with relative humidity (RH) (Figure 1d; see Figure S1 in the Supporting Information for details). This distinct color change of the phage layer on HLRP is realized by strong resonant absorption at a specific wavelength due to a sensitive resonance change compared to that cases on other substrates (Figure 1e; see Figure S2 in the Supporting Information for details).

**Figure 1:**
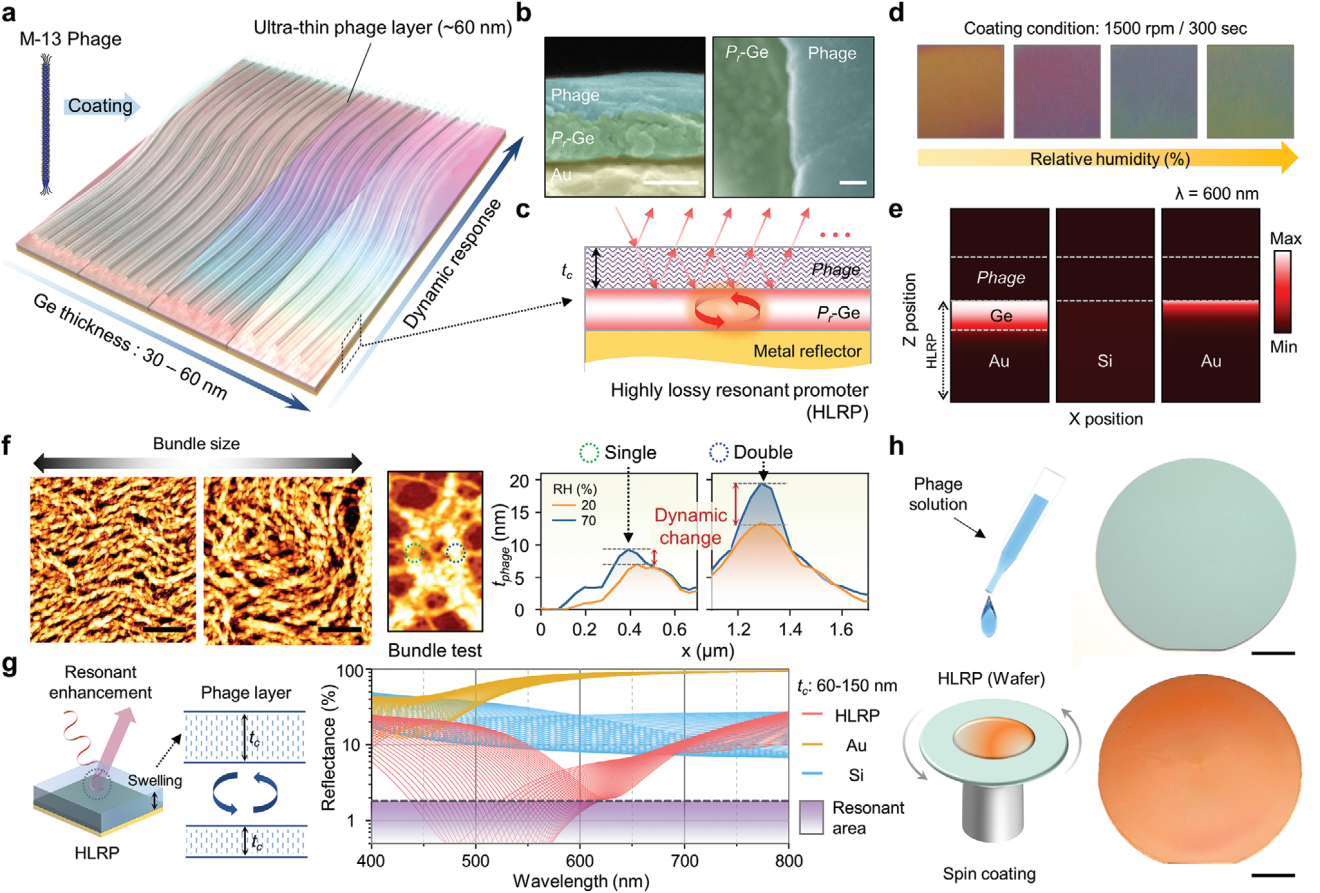
a) Schematic illustration of colorimetric sensor with corresponding Ge thickness and relative humidity with M‐13 phage coating. b) SEM image showing phage‐coated porous Ge layer of cross‐sectional (left) and top view SEM image (right). Scale bar is 100 nm. c) Schematic showing highly lossy resonant promoter (HLRP) for resonance enhancement. d) Color images of colorimetric sensor corresponding to different humidity levels. e) Absorption intensity distributions of phage‐coated HLRP, Si, and Au, respectively. f) AFM images and thickness level profiling showing bundle size change by humidity conditions. Scale bar is 5 µm. g) Schematic illustration of resonant enhanced color reflection mechanism with phage layer swelling and reflectance spectra showing enhanced absorption and dip shift. h) Schematic of spin‐coating method and sample images onto 2‐inch wafer before (top) and after (bottom) phage spin‐coating. Scale bar is 1 cm.

For surface analysis of the dynamic responsive layer, Figure 1f shows the dynamic response characteristics for the phage bundle. The bundle size of the phage layer is varied with spin‐coating conditions related to shear force and solution concentration (Figure 1f left; see Figure S3 in the Supporting Information for details).^[^ ^31^ ^]^ For rigorous testing of the dynamic range of ultrathin phage layers, the test sample formed locally in single‐ and double‐layers of phage bundles was measured for the dynamic change with water adsorption using atomic force microscopy (AFM) (Figure 1f right; see Figure S4 in the Supporting Information for details).^[^ ^32^ ^]^ The result shows a drastic dynamic change with water adsorption in the double layer of the phage bundle. To confirm the resonance enhancement of the HLRP with the phage layer, reflectance spectra were calculated for dynamic changes in the HLRP and other substrates (Figure 1g). At a wavelength range of 500–600 nm, in which the spectral response of the color is sensitive, depending on the tristimulus function,^[^ ^33^ ^]^ the HLRP shows a series of resonance dips closer to zero reflection than for other substrates with dynamic changes. For scalability of the proposed scheme, a spin‐coating method was exploited to form a large area ultrathin phage layer with uniformity. A large area ultrathin phage layer was uniformly coated under optimal coating conditions, including solution concentration and rotational speed, on the wafer‐scale HLRP prepared by a glancing angle deposition using an e‐beam evaporator (Figure 1h).^[^ ^34^, ^35^, ^36^ ^]^

For optimal design of the HLRP, **Figure** **2**a shows a contour map of the calculated reflectance versus the complex refractive index at a specific wavelength (*λ*_c_ = 500 nm), along with a schematic of the composition of each separated HLRP layer. For the computational model, a coating layer with a measured refractive index was constructed on a resonance promoter consisting of a lossy medium and a metal reflector (Figure S5, Supporting Information). From the 3D reflectance surface, the resonant area was defined by projecting the purple resonant dip onto 2D space as a function of the complex refractive index. Lossy media satisfying such a resonant area can be realized by controlling the porosity (*P*_r_) of the highly absorbent material. The close proximity of the resonance dip to zero reflection by the HLRP can be a key consideration in improving chromaticity, as shown in Figure 2b. The calculated result of color saturation with the reflectance position of the resonant dip shows a drastic enhancement of chromaticity by triggering a spectrally selective response of the tristimulus color matching function as the dip approaches zero reflection (see Figures S6 and S7 in the Supporting Information for details). Depending on the dynamic change of the coating layer, these reflective dips with HLRP exhibit resonant wavelength shifts in the visible range, causing colorimetric behavior (Figure 2c).

**Figure 2:**
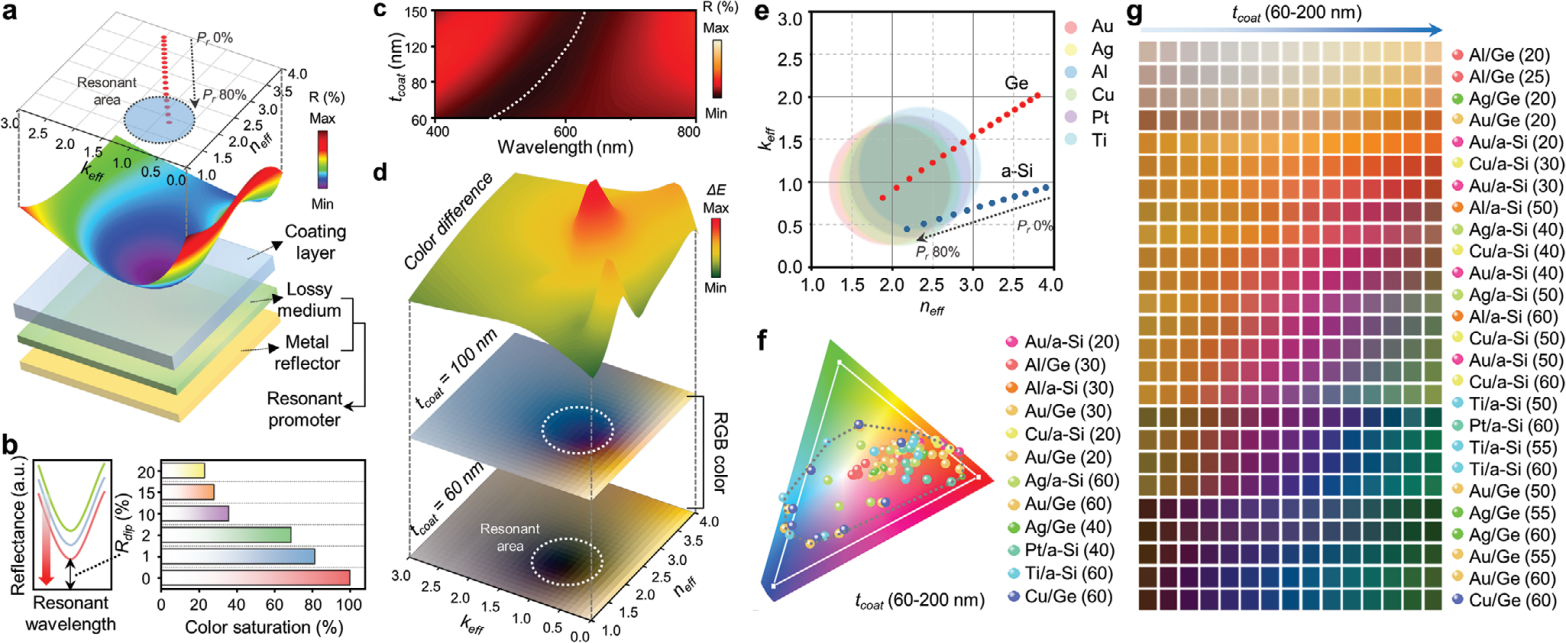
a) Contour map of the calculated reflectance versus complex refractive index at a specific wavelength (*λ*_c_ = 500 nm), along with a schematic of the composition of each separated HLRP layer. b) Color saturation of each reflectance spectrum having different dip reflectances (*R*_dip_) (*λ*_dip_ = 500 nm). c) Reflectance contour with dynamic change of coating layer on the HLRP of Au/Ge (*P*_r_ 75%, *t*_Ge_ = 60 nm). d) RGB color representations and corresponding color differences (Δ*E*) with different coating layers on 2D surface of complex refractive index. e) Complex refractive index coordinate, which contains resonant area for various materials. As *P*_r_ increases, the complex refractive index of HLRP converges to the resonant area. f) Chromaticity plots for sRGB gamut (white line) on CIE coordinates, indicating the colors of phage‐coated HLRP for various material and thickness combinations. g) Color palette of several material and thickness combinations with dynamic change (*t*_coat_ 60–200 nm).

To confirm the chromatic response with the dynamic change, Figure 2d presents RGB color sets with consideration of each coating layer of 60 nm, as the initial thickness of the spin coated virus layer, and 100 nm. As expected from the tendency of the reflectance dip, the results show distinct color changes with high color differences only near the resonant area indicated by the white dashed line, whereas the remainder of the area exhibits only a very subtle difference in the color of the metal reflector. Following our design process of the resonant promoter, resonant areas of various metal reflectors were plotted on 2D coordinates of the complex refractive index, as depicted in Figure 2e (see Figure S8 in the Supporting Information for details). The combination of materials with the resonant areas superimposed by the *P*_r_ control of the highly absorbent materials enables the design of HLRP, which allows distinct color changes with the dynamic responsive layer. For wide color selectivity with dynamic change, chromatic values of material combinations derived from the previous results with resonant areas were plotted using the sRGB color gamut on CIE coordinates (Figure 2f; see Figure S9 in the Supporting Information for details). Depending on the material combination of the resonance promoter, chromaticity values are widely or locally varied with dynamic changes within the sRGB color gamut. Converted from these chromatic values, Figure 2g shows a color palette with wide color selectivity for various material combinations (see Figure S10 in the Supporting Information for details). These color sets provide the selective chromatic responsivity of the proposed colorimetric sensor to allow for sensitive/insensitive design to external stimuli.

For experimental confirmation as a representative material combination of the HLRP, we conducted an optimization of the fabrication process with different concentrations (11–15 mL mg^−1^) of phage solution using Au/Ge combinations, as illustrated in **Figure** **3**a. Depending on the concentration of phage solution, the bundles showed different sizes on the surface of the HLRP, resulting in a different degree of expansion by swelling and consequently causing several color response sensitivities. For humidity sensing, with increasing concentrations of phage solution, HLRPs were regularly spin‐coated to satisfy sensitive color responses over a wide range (20–90%) of relative humidity, as observed in the humidity measurement setup (see Figure S11 in the Supporting Information for details). Moreover, as expected from the calculation results, HLRPs of different thicknesses (30–60 nm) coated with the optimized phage concentration (15 mg mL^−1^) exhibit selective chromatic responsivities (see Figure S12 in the Supporting Information for details). For quantitative evaluation in terms of chromaticity, Figure 3b shows the hue angle rotation and *Δ*Hue length converted from the measured reflectance of phage‐coated HLRPs (see Figure S13 in the Supporting Information for details). With different thicknesses and solutions, the phage‐coated HLRPs exhibited the highest hue length (288°) under the optimal conditions (60 nm and 15 mg mL^−1^), in which both the resonant and dynamic responsivity were harmonized over a wide range of humidity environments. (see Figure S14 in the Supporting Information for details).

**Figure 3:**
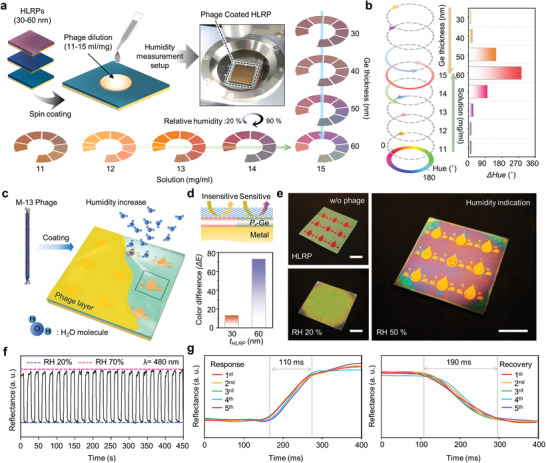
a) Schematic illustration showing colorimetric sensing optimization process according to phage dilution and absorbing layer (*P*_r_‐Ge) thickness with reflectance spectra measurements. b) Degree of hue angle change of various conditions from (a). c) Schematic illustration of colorimetric sensor display with visualization process insensitive/sensitive color difference. d) Regional design of insensitive/sensitive area with different chromatic response (top); color difference (Δ*E*) of insensitive/sensitive area (bottom). e) Color images of colorimetric sensor display in different states (before/after phage coating, and humidity indicating state, respectively). Scale bar is 1 cm. f) Reproducibility test result and g) response/recovery time measurement result by repeated exposure to RH 20% and 70%.

As a practical demonstration of selective chromatic responsivity, Figure 3c shows a schematic of the humidity indicator with hidden/revealed patterns that are dependent on the humidity environment. The pattern area was designed to be spatially insensitive/sensitive to humidity changes (Figure 3d top; Figure S15, Supporting Information). With different HLRP thicknesses, each chromatic responsivity was confirmed as a color difference (∆*E*) with humidity (Figure 3d bottom; Figure S16, Supporting Information). Figure 3e shows sample images of a droplet‐patterned humidity indicator fabricated by a conventional lithographic process. Without a phage coating, HLRPs of different thicknesses in each pattern area have their own distinct colors (Figure 3e upper‐left). However, phage coated HLRPs show a similar color at low humidity in both areas, as the pattern is designed to be hidden (Figure 3e bottom‐left; see Figure S17 in the Supporting Information for details). With the dynamic change of the phage layer at high humidity, the responsive color of the insensitive area rarely changes, while a distinct color change is realized in the sensitive area, revealed in a droplet pattern with angle robustness enabling reliable observation in everyday life (Figure 3e right; see Figures S18 and S19 in the Supporting Information for viewing‐angle independency). Furthermore, due to the optimized ultrathin phage layer, the colorimetric humidity indicator displays a rapid response to drastic changes in humidity, such as breath blowing. After the moisture disappears, the fabricated indicator is reversibly returned to its initial hidden pattern with the deswelling of phage layer. (Movies S1 and S2, Supporting Information). For reproducibility test, the phage coated HLRP was repeatedly exposed to RH 20% and 70%, cyclically. As shown in Figure 3f, the reflectance was reversibly fluctuated (*λ* = 480 nm) between ambient condition (RH 20%) and humid state (RH 70%). Moreover, to quantitatively confirm responsiveness, response/recovery times were also measured. As shown in Figure 3g, as a rapid response, the measured response/recovery times are 110 and 190 ms at RH 20% and 70%, respectively. These results show a comparable response and duration with nanomaterial‐based resistive and/or capacitive sensors.^[^ ^37^ ^]^

As a diverse substances detection, **Figure** **4**a illustrates the wild type (WT) phage with three genetically engineered type (3A, 4E, and 3W) phage and shows a series of sensing process with multicolorimetric sensor array (MCSA). Each pixel coated with four different phage showed noticeable color variations to subtle environmental change even in ppb level beyond the ppm scale due to the sensitive color response of HLRP. To analyze these color responses, the RGB values of MCSA were detected with varying concentration of VOCs (i.e., acetone, isopropyl alcohol, diethyl ether, and benzene) and EDCs (i.e., diisobutyl phthalate (DiBP) and di‐*n*‐butyl phthalate (DnBP)) under enclosed condition (see Figure S20 in the Supporting Information for detail setup). Each genetically engineered phage has a selective response to different substances by the interaction characteristics of each receptor (see Figure S21 in the Supporting Information for details). With these in‐built responses of genetically engineered phages, each combination of RGB values corresponding to concentration and material species forms a unique data set. For intuitive comparison, RGB values converted into color difference (Δ*E*) and *Δ*RGB intensity (see Figure S22 in the Supporting Information for colorimetric calculation results in detail). For selectivity of MCSA for various substances, Figure 4b depicts fingerprint patterns by combinations of selective chromatic responses to different substances for each genetically engineered phage. These fingerprint patterns based on Δ*E* showed particular shapes for each substance even in chemically similar material such as DiBP, DnBP, and benzene. As a result of quantitative measurements for sensitivity of MCSA, color palettes based on *Δ*RGB intensity also have different color patterns corresponding to each chemical substance at ppb scale concentration (Figure 4c; see Figure S23 in the Supporting Information for details).

**Figure 4:**
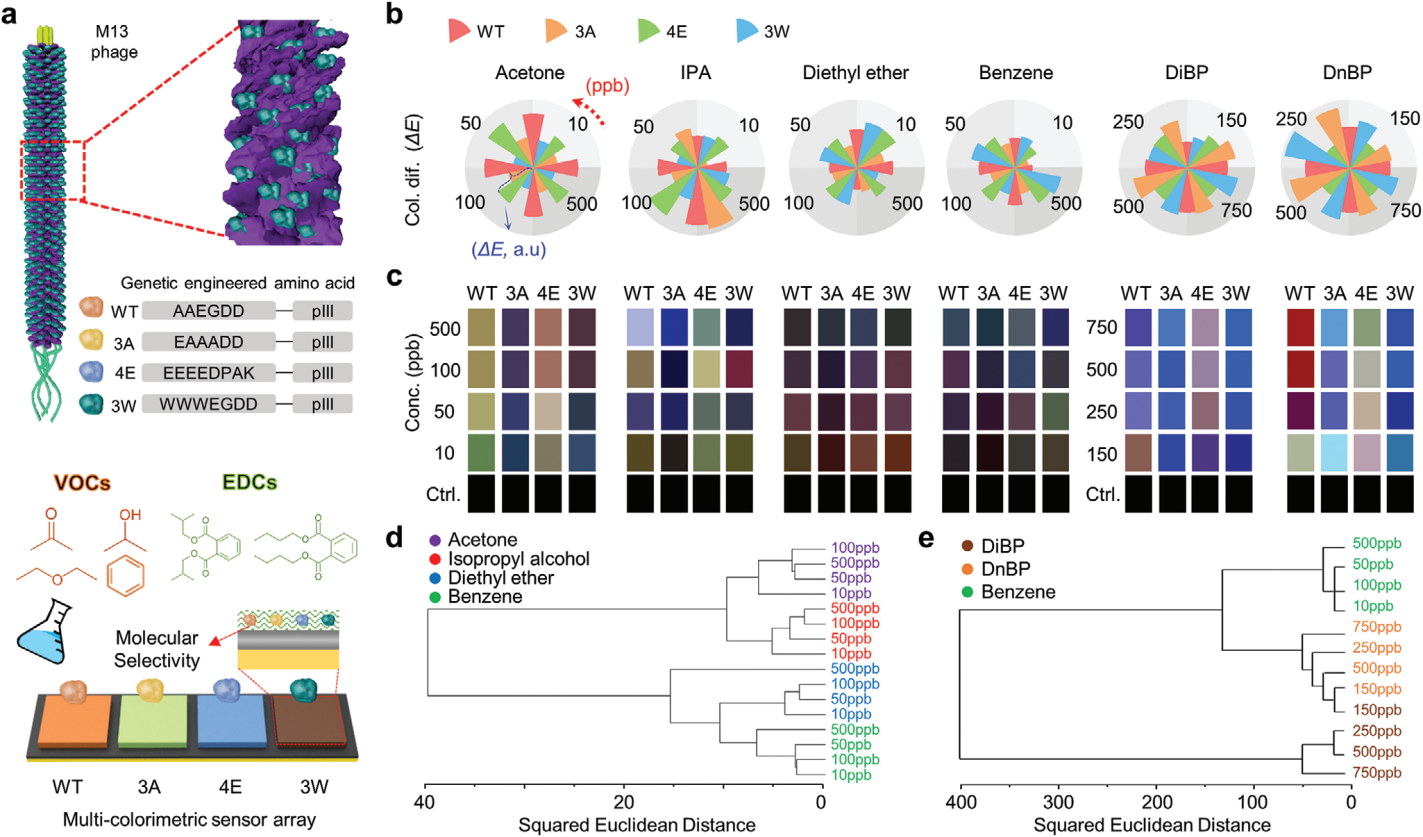
a) Schematic illustration of genetically engineered M‐13 phage and engineered sequences (top). Schematic of multicolorimetric sensor array (MCSA) with four engineered phage coating layer (WT, 3A, 4E, and 3W) on HLRP (bottom) under volatile organic compounds (VOCs) (acetone, isopropyl alcohol, diethyl ether, and benzene) and endocrine disrupting chemicals (EDCs) (diisobutyl phthalate (DiBP) and di‐*n*‐butyl phthalate (DnBP)) b) Fingerprint patterns obtained by color difference of MCSA with varying chemical substance and concentration change. c) Color palette based on *Δ*RGB intensity with varying chemical substance and concentration change. Hierarchical cluster analysis (HCA) of d) VOCs and e) EDCs/benzene in accordance with different concentrations.

For a quantitative classification, hierarchical cluster analysis (HCA) were performed as a statistical classification method using the acquired *Δ*RGB intensity data from four steps concentrations and six chemical substance species (i.e., VOCs and EDCs). To evaluate the distance information, the closest data were classified into the same cluster based on Euclidean distance to indicates a similarity of each object. As shown in Figure 4d, the hierarchy was well classified according to the type of substances, showing VOCs classified at even small concentration of 10 ppb. In particular, Figure 4e shows the EDCs were successfully differentiated to 150 ppb lower than the 5 mg m^−3^, which is permissible exposure limits specified by the Occupational Safety and Health Administration (OSHA) (see Table S1 in the Supporting Information for details).^[^ ^38^ ^]^ Furthermore, following these results, EDCs and benzene, which is generally used as a solvent for DiBP and DnBP were also well separated. Furthermore, for confirmation of the reversibility of MCSA, three times measurement were performed and output colors show similar results, and the similarity was confirmed with PCA plots (see Figure S24 in the Supporting Information for details).

We presented an effectual strategy for intuitively detecting environmental changes using a strong resonant structure with geometrical simplicity and highly selective dynamic responsive material. An ultrathin M‐13 phage layer, spin‐coated over an HLRP, displays sensitive colorimetric behavior with strong resonance changes for a rapid dynamic response. Considering the complex refractive index changes, the responsive chromaticity was improved by optimizing the resonance area for the HLRP design, and wide chromatic responsivity was verified by performing the optimization process for various material combinations. For practical applications, we demonstrated a colorimeter sensor, indicating humidity detection by revealing spatially designed droplet patterns with selective chromatic responsivity. Moreover, hazardous chemical detection using genetically engineered phage and large‐area fabrication using the spin coating method open up the potential for expanded applications in everyday and industrial environments. Tailoring simple photonic structures into ultrathin dynamic responsive material, the proposed design is an attractive converging technology for selective colorimetric sensing and hazardous substance detection.

## Experimental Section

##### Optical Calculation

RCWA was used to calculate the reflectance of the phage coating films on the HLRP and other substrates using commercial software (DiffractMOD, RSoft Design Group, USA). In the RCWA, the simulation condition was set to the second diffraction order and 0.2 nm grid size to calculate the diffraction efficiency, which was enough to numerically stabilize the results. In addition, to obtain accurate output, material dispersions and extinction coefficients were considered. The commercial software MATLAB (MathWorks, USA) was also used to calculate both the effective complex refractive indices based on volume averaging theory (VAT),^[^ ^39^ ^]^ and the chromatic information from the reflectance.^[^ ^33^ ^]^

##### Optical Characterization

The reflection spectra of all fabricated samples were measured with a UV–vis–NIR spectrometer using a tungsten‐halogen lamp light source at a normal incidence angle. To measure the reflectance according to humidity change, N_2_ gas was passed through the humidifier to contain moisture; it was then injected into the chamber with matching between humidity and reflectance spectra change. The optical constant was obtained using an ellipsometer (RC2, J.A. Woollam Co., USA) with a He–Ne laser as a source.

##### Preparation of Genetically Modified Bacteriophages M‐13

The major protein (pVIII) of WT phage was modified into A(Ala)A(Ala)A(Ala), E(Glu)E(Glu)E(Glu)E(Glu), and W(Trp)W(Trp)W(Trp) using site‐directed mutagenesis PCR techniques.^[^ ^28^ ^]^ Using this primer set, the partial target sequence of the form was designed with 3A (hAAA), 4E (EEEE), and 3W (WWW). The antisense primer and sense primer were designed to make the insertion peptide linear and complementary to the engineered gVIII region. To incorporate the gene sequences, PCR was amplified using Pfu DNA polymerase, two primers (insertion and linearization), and an M13KE (NEB) vector as the template. The obtained product was incubated Dpn1 and transformed into XL1‐Blue competent bacteria cells. The amplified plasmid sequence was verified with a DNA sequencing facility (Bionics Co., Korea). The constructed phages were amplified by bacterial cultures and purified, sequentially, concentrated through polyethylene glycol precipitation.

##### Preparation of Phage Coated HLRP

HLRP ultrathin films were fabricated by glancing angle deposition (GLAD) to achieve a porous medium. As a substrate, single‐side polished silicon (100) wafers was selected, and was treated with buffered oxide etchant to remove the native oxide layer. Sequentially, each was sonicated in the order of acetone, methanol, and DI water for 5 min, respectively. Both the metal reflector (Au) and absorbing layer (Ge) of HLRP were deposited by electron beam evaporation (KVE‐E2000, Korea Vacuum Tech Co., Korea) under a high vacuum (≈10^−6^ Torr). The Au film was deposited at a rate of ≈2 Å s^−1^ to a thickness of 100 nm, which is sufficient as a metal reflector. The porous Ge layer was deposited at a rate of ≈2 Å s^−1^ to target thickness on the Au film after mounting the substrate on a tilted sample holder (customized). To ensure uniformity, deposition was performed until half the target thickness was reached, then the e‐beam flux was stopped, and the sample was reloaded upside‐down 180° and deposition was restarted.^[^ ^34^ ^]^ At this point, the tilted sample holder was kept facing the Ge source in the same direction as the first deposition to form a porous medium. To coat the phage layer, the prepared phage solution was dropped onto the HLRP and spin‐coated at 1500 rpm for 300 s.

##### Fabrication of Colorimetric Sensor Display

To pattern the colorimetric sensor display, photolithography was used with image reversal photoresist (AZ 5214, AZ Electronic Materials, Luxembourg). For the image reversal process, a mask aligner (MJB3 UV400, Karl Suss, Germany) was used with the patterned photo mask. In each patterned area, Ge layers were deposited with different thicknesses to achieve insensitive/sensitive responses; the lift‐off process was then performed to reveal each patterned area.

##### Measurement Process of MCSA

Each part of the MCSA was fabricated with phage spin‐coating (WT, 3A, 4E, and 3W) on the HLRP layer. The concentrations of VOCs and EDCs were controlled by increasing the temperature of the chamber. Simultaneously, the real‐time color change of the MCSA was captured by a digital microscope (Celestron, USA) every 5 s. In each capture, the RGB value was extracted and the *Δ*RGB intensity and color difference (Δ*E*) was calculated based on the initial state color of the MCSA for each pixel. To meet the steady state at the target concentration, measurements were performed for 3 min.

## Conflict of Interest

The authors declare no conflict of interest.

## Supporting information

Supporting Information

Supporting Information

Supporting Information
