# Supplementary material for: Large‐Area Virus Coated Ultrathin Colorimetric Sensors with a Highly Lossy Resonant Promoter for Enhanced Chromaticity
Source: Adv Sci (Weinh). 2020 Jul 21;7(18):2000978. doi: 10.1002/advs.202000978 (PMC7509654; doi:10.1002/advs.202000978)
Supplement: Supplementary file 1 — Supporting Information [file ADVS-7-2000978-s001.pdf]

Copyright WILEY-VCH Verlag GmbH & Co. KGaA, 69469 Weinheim, Germany, 2020.

ADVANCED SCIENCE
Open Access

Supporting Information

for *Adv. Sci.*, DOI: 10.1002/advs.202000978

Large-Area Virus Coated Ultra-Thin Colorimetric Sensors with a Highly Lossy Resonant Promoter for Enhanced Chromaticity

Young Jin Yoo†, Won-Geun Kim†, Joo Hwan Ko†, Yeong Jae Kim, Yujin Lee, Stefan G. Stanciu, Jong-Min Lee, Seungchul Kim, Jin-Woo Oh, and Young Min Song**

Supporting Information 1: Color measurements of fabricated samples.

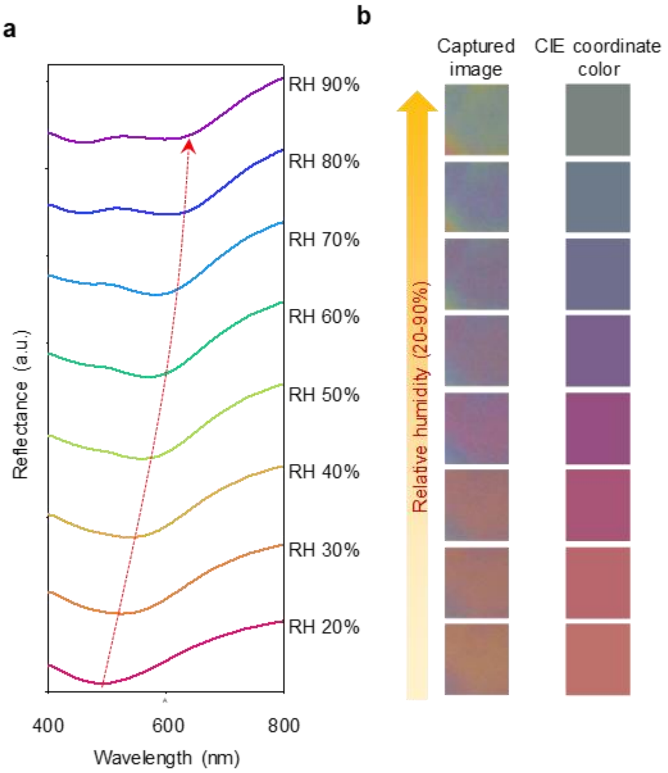

Figure S1. a) Measured reflectance spectra of phage coated highly lossy resonant promoter (HLRP) with humidity change (RH 20−90%). b) Captured image of phage coated HLRP and CIE coordinate color for prepared with 15 mg/ml phage solution at 1500 rpm.

Supporting Information 2: Comparison with different substrates.

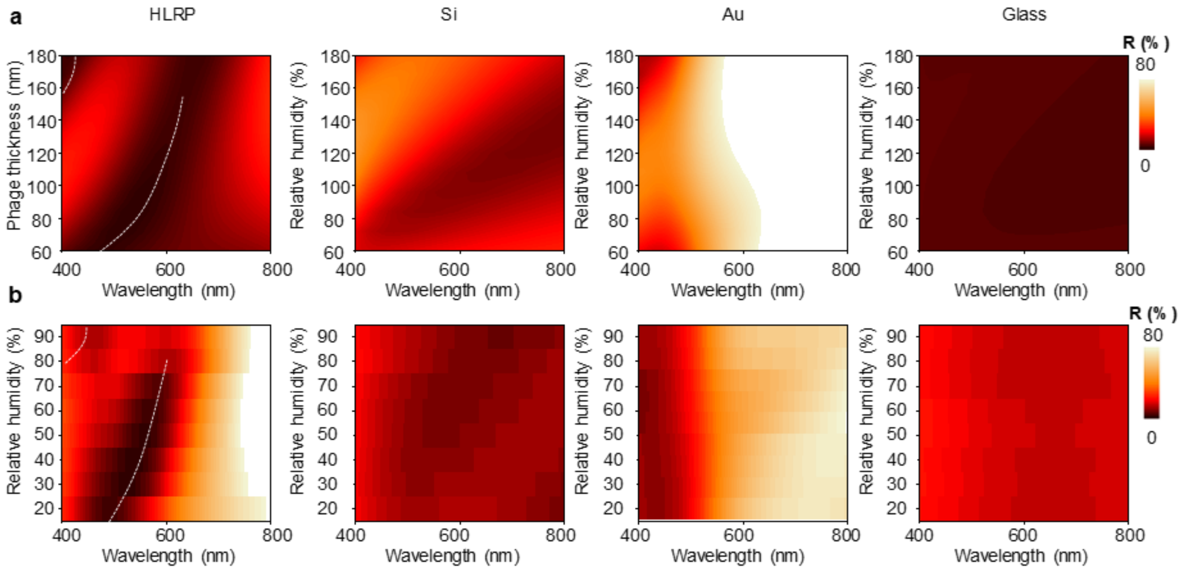

Figure S2. a) Simulated reflectance contour and b) measured reflectance contour for phage coated various substrates (HLRP, Si, Au, and glass, respectively). White dashed lines represent the minimum dip position shift. Based on each dip position comparison, the humidity level and thickness of phage (t_{phage}) was matched.

Supporting Information 3: Phage coating surface analysis.

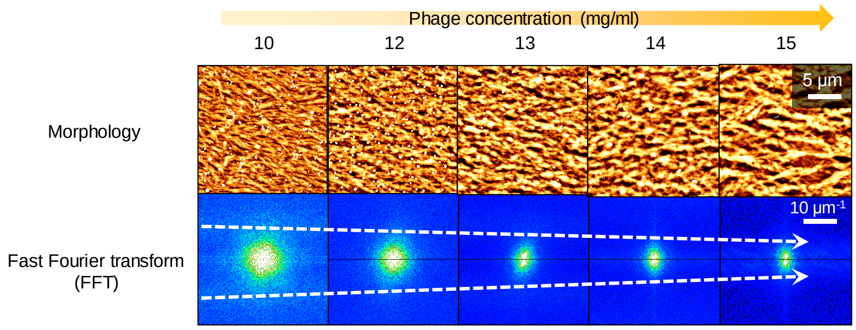

Figure S3. AFM images and fast Fourier transform (FFT) patterns of the M-13 phage films for each phage solution concentration at 1500 rpm of spin coating rate. According to the FFT analysis, as the concentration of the M-13 phage solution increasing, the bundle size and bundle-to-bundle distance becomes thicker and longer, respectively.

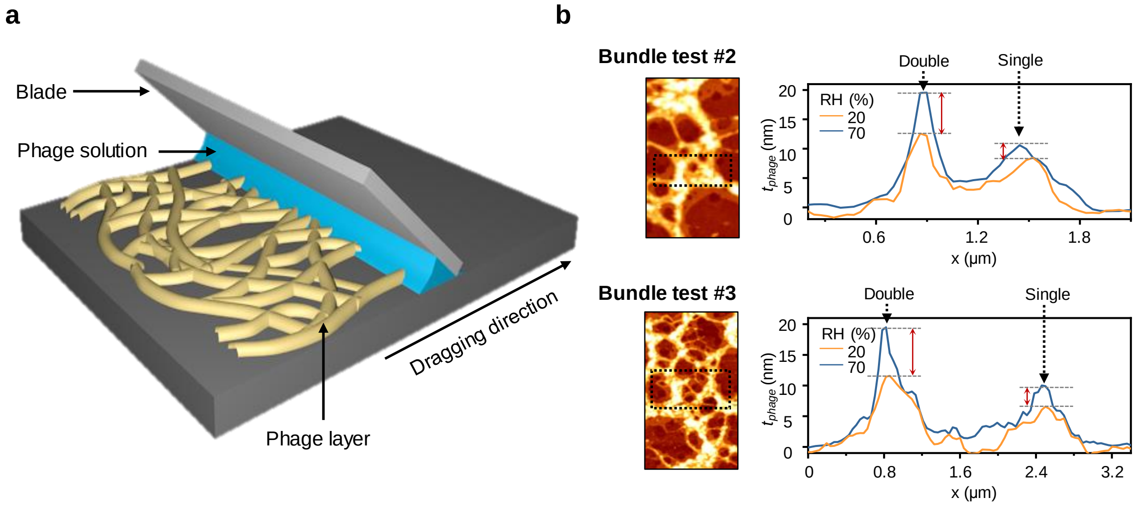

Figure S4. a) Schematic image of phage deposition process by meniscus-dragging deposition (MDD) method.[S1] b) AFM images and thickness level profiling showing bundle size change by humidity conditions for three different films deposited by MDD.

Supporting Information 4: Ellipsometry measurement of phage coating layer.

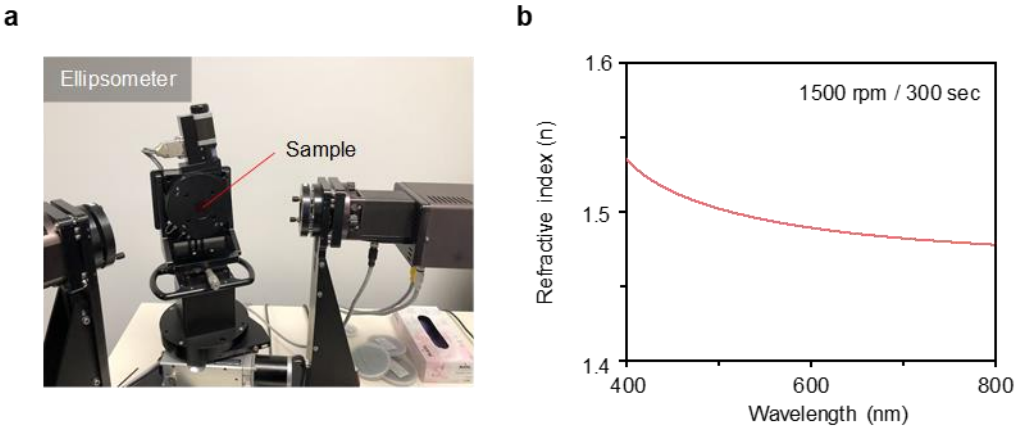

Figure S5. a) Ellipsometry measurement setup. b) Measured refractive index of the phage layer by spin coating at 1500 rpm for 30 sec.

Supporting Information 5: Chromatic analysis.

The Color information is expressed through tristimulus values, with light intensity based on three basic color values (RGB), usually expressed in X, Y, and Z coordinates, respectively. To convert from the reflectance spectrum to color information, the tristimulus value is calculated as

$$X = \int M(\lambda)\bar{x}(\lambda)d\lambda, \quad Y = \int M(\lambda)\bar{y}(\lambda)d\lambda, \quad Z = \int M(\lambda)\bar{z}(\lambda)d\lambda\,, \qquad (1)$$

where $\bar{x}(\lambda), \bar{y}(\lambda), and\ \bar{z}(\lambda)$ are color matching functions, which are the numerical description of the chromatic response of the observer; and $M(\lambda)$ is the reflectance or transmittance over the customary limited range (400–800 nm). In this study, we applied the CIE 1931 color space, which is the most widely used, for evaluating the color information from measured or simulated reflectance spectra. The chromaticity is specified by two derived parameters x and y, which indicate the spectral selectivity of the relative response of the tristimulus values to describe the CIE coordinates:[S2, S3]

$$x = \frac{X}{X+Y+Z} \qquad y = \frac{Y}{X+Y+Z} \quad . \qquad (2)$$

Furthermore, the tristimulus values can also be converted into Red, Green, and Blue (RGB) color expression values by using the simple matrix given by

$$\begin{bmatrix} R \\ G \\ B \end{bmatrix} = \begin{bmatrix} 0.41847 & -0.15866 & -0.082835 \\ -0.091169 & 0.25243 & 0.015708 \\ 0.00092090 & -0.0025498 & 0.17860 \end{bmatrix} \begin{bmatrix} X \\ Y \\ Z \end{bmatrix} \quad . \qquad (3)$$

In this study, we calculated Equation 1, Equation 2, and Equation 3 using MATLAB.

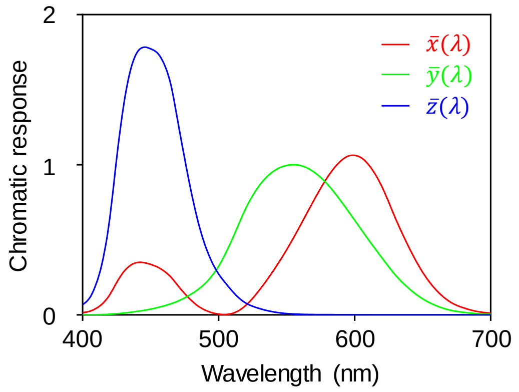

Figure S6. Chromatic response of the tristimulus function from CIE 1931 standard observer.[S2]

Supporting Information 6: Comparison of the chromaticity and dip position.

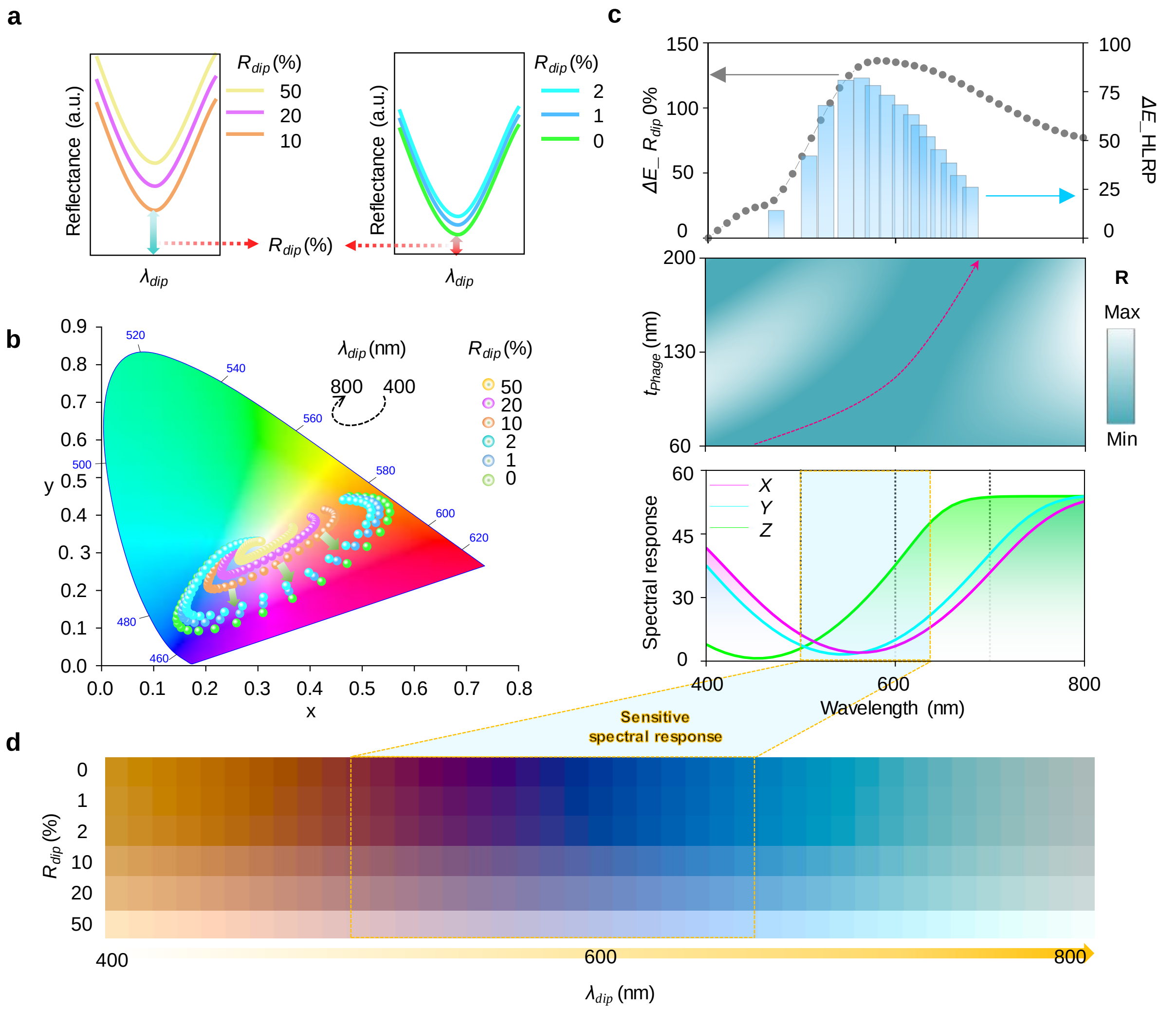

Figure S7. a) Reflectance spectra with various dip reflectance (R_{dip}) obtained by Gaussian distribution. b) CIE coordinates by dip position (λ_{dip}) shift with various range R_{dip} (0–50%). c) Color difference (*ΔE)* with the dip shift of R_{dip} 0% and phage coated HLRP (top) and reflectance contour with dip shift of phage coated HLRP to t_{coat} (thickness of phage coating layer) increases (middle). Spectral responses of tristimulus function with dip position shift (400–800 nm) of R_{dip} 0% spectrum. The response of Z shows drastic change in λ_{dip} 500–650 nm (bottom).[S2] d) Color palettes of each R_{dip} with dip position shift (400–800 nm) with sensitive color change λ_{dip} 500–650 nm.

Supporting Information 7: Optimization calculations with various complex refractive indices.

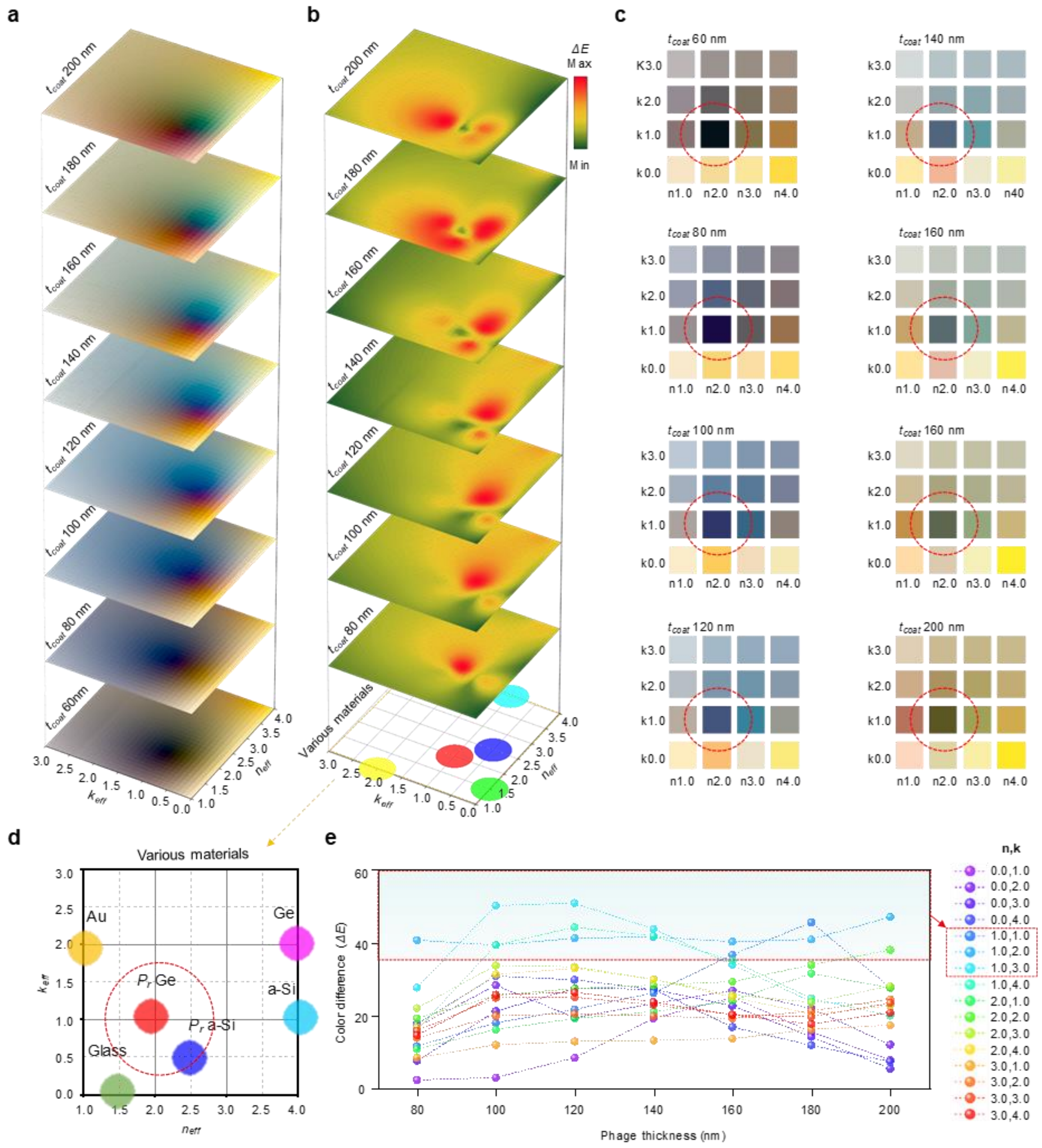

Figure S8. a) Color palettes of the HLRPs with different coating thickness (t_{coat}) according to complex refractive index. b) Color difference (ΔE) contours of calculated results by thickness change (t_{coat} = 80–200 nm) from initial coating layer (t_{coat} = 60 nm). c–e) The dashed red circles and rectangle indicate the conditions with clear colors and large color differences. c) Selected

color palettes from a). d) Complex refractive index coordinate with various materials. Each P_r Ge and P_r a-Si was calculated with P_r 75%. e) Color differences (ΔE) from the colors in c).

Supporting Information 8: CIE coordinates with universal selectivity.

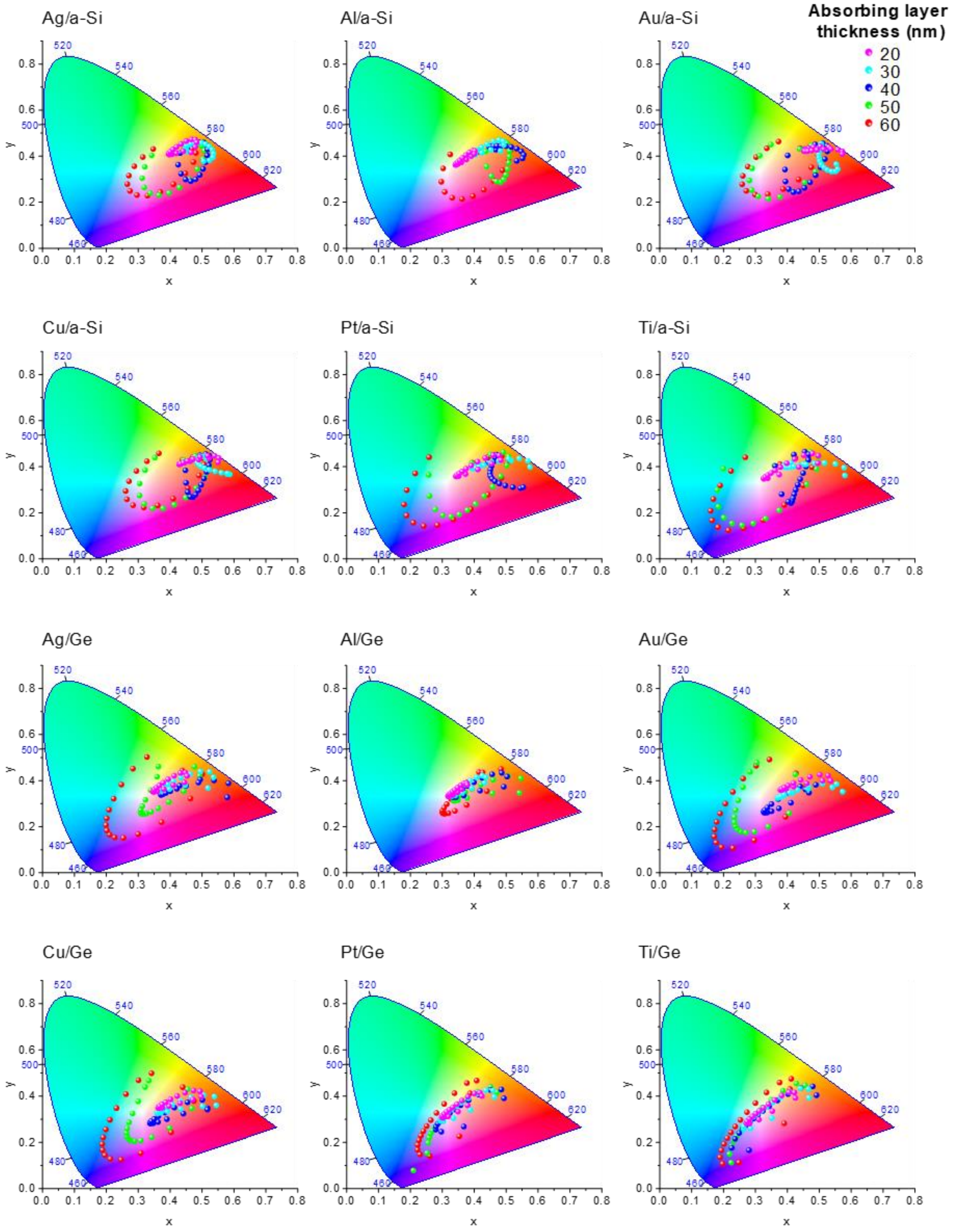

Figure S9. CIE coordinates of phage coated HLRP films (t_{coat} 60–200 nm) for several material and thickness combinations.

Supporting Information 9: Color expression with universal selectivity.

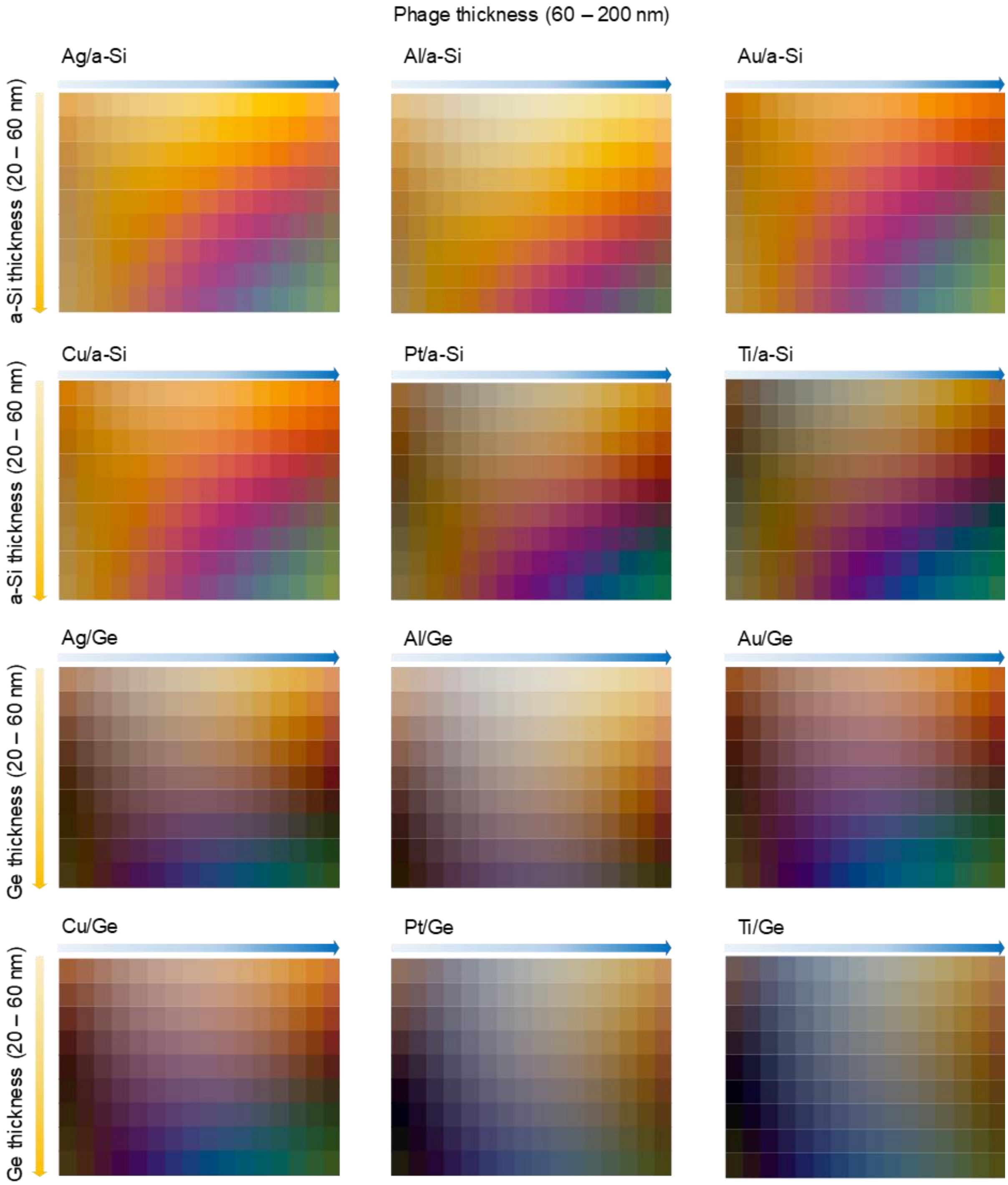

Figure S10. Color expression of phage coated HLRP films (t_{coat} 60–200 nm) for several material and thickness combinations.

Supporting Information 10: Measurement setup

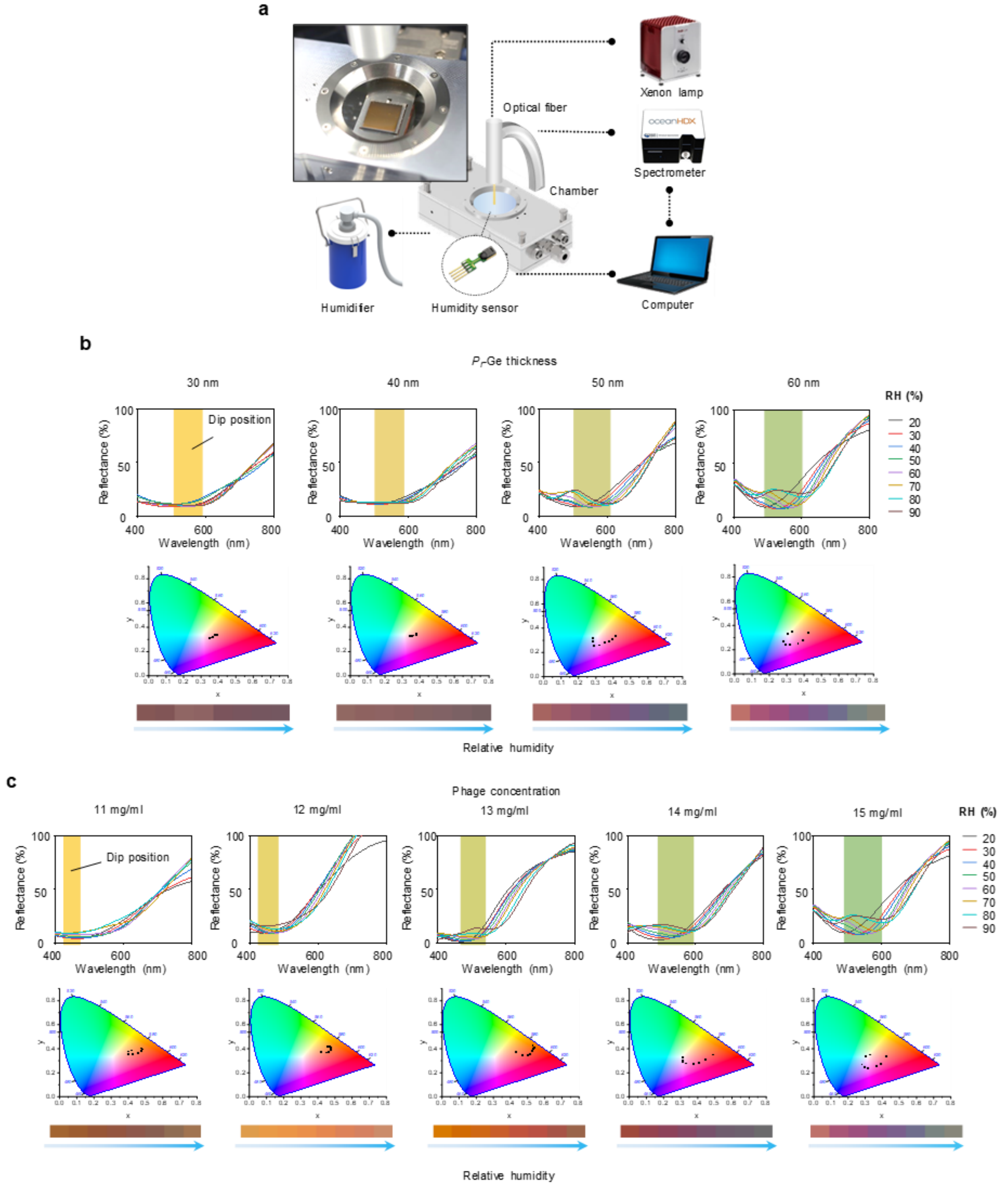

Figure S11. a) Schematic diagram of optical measurement setup with a humidifier, humidity sensor, and gas chamber. b) Measured reflectance spectra, CIE coordinates, and color expression to humidity changes (RH 20−90%). Concentration of the phage solution is 15 mg/ml. c) Measured reflectance spectra, CIE coordinates, and color expression to humidity changes (RH 20−90%).

Supporting Information 11: Reflectance and color with different HLRPs.

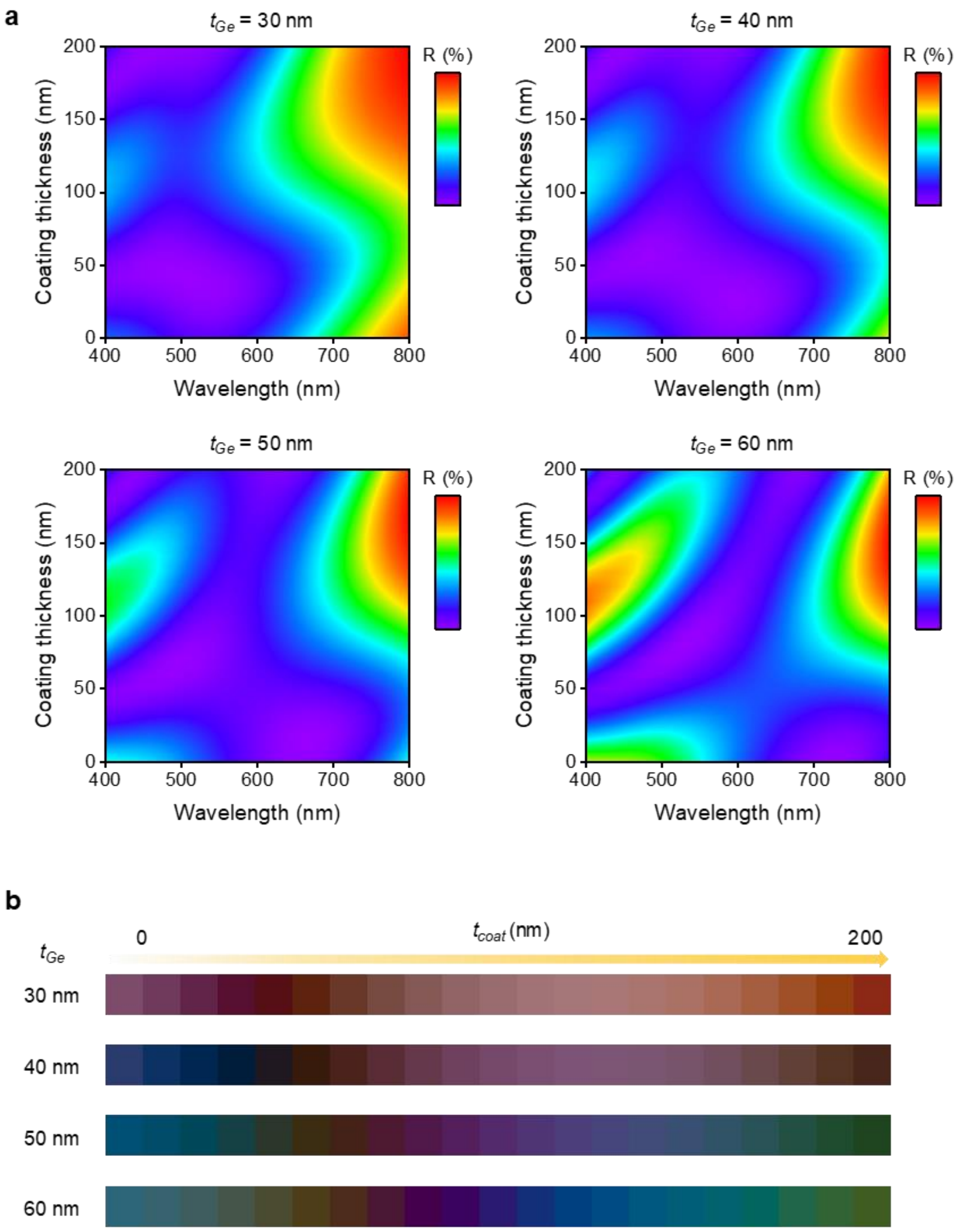

Figure S12. a) Calculated reflectance contours for each thicknesses of HLRP (t_{Ge} = 30–60 nm) with coating layer thickness change (t_{coat} = 0–200 nm). b) Color representation of a).

Supporting Information 12: Hue angle rotation of samples with different concentrations and thicknesses.

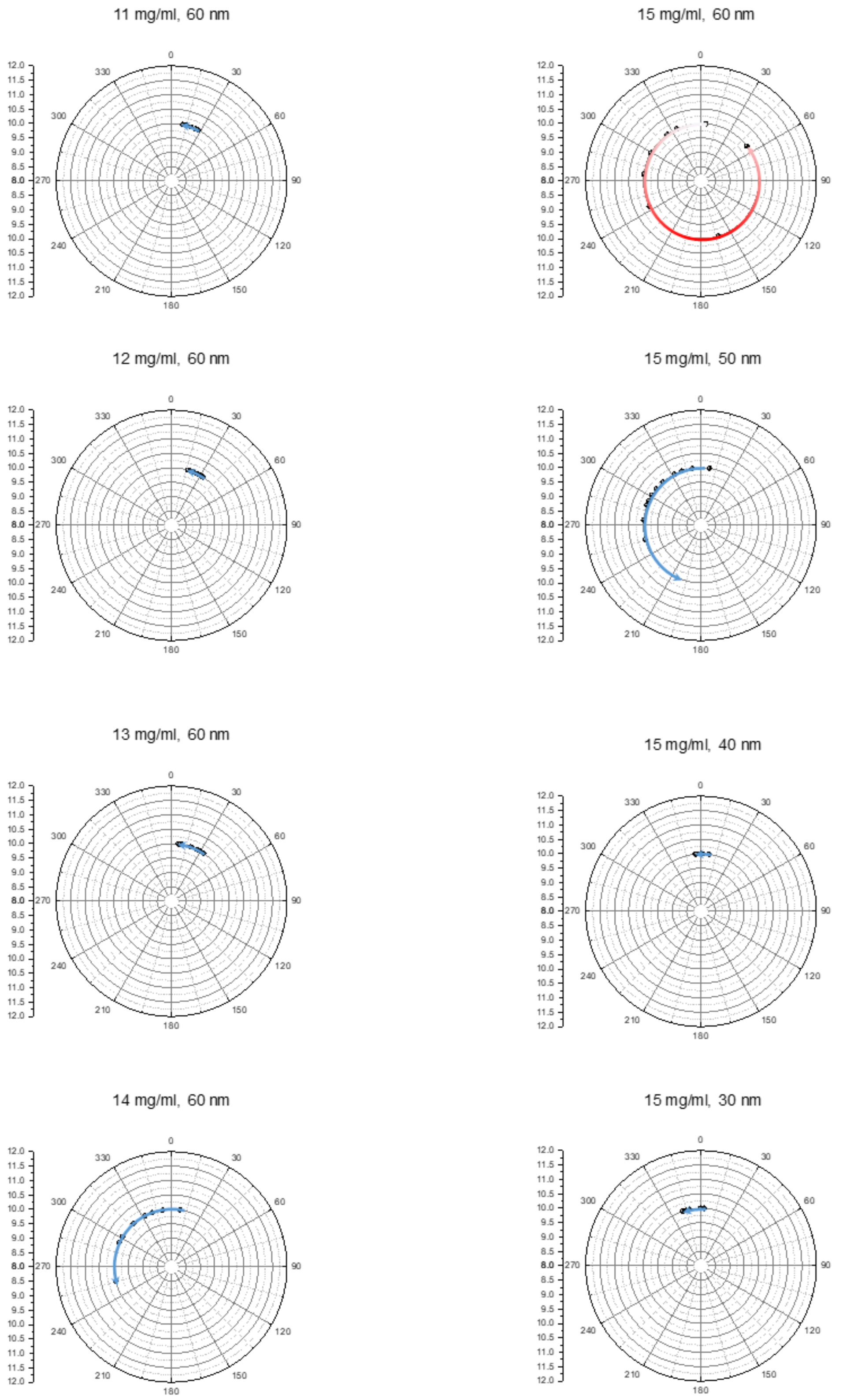

Figure S13. Hue angle plots converted from the measured reflectance of different samples with different phage solution concentration (11–15 mg/ml) and HLRP thicknesses (30–60 nm).

Supporting Information 13: Optical measurement and colorimetric analysis.

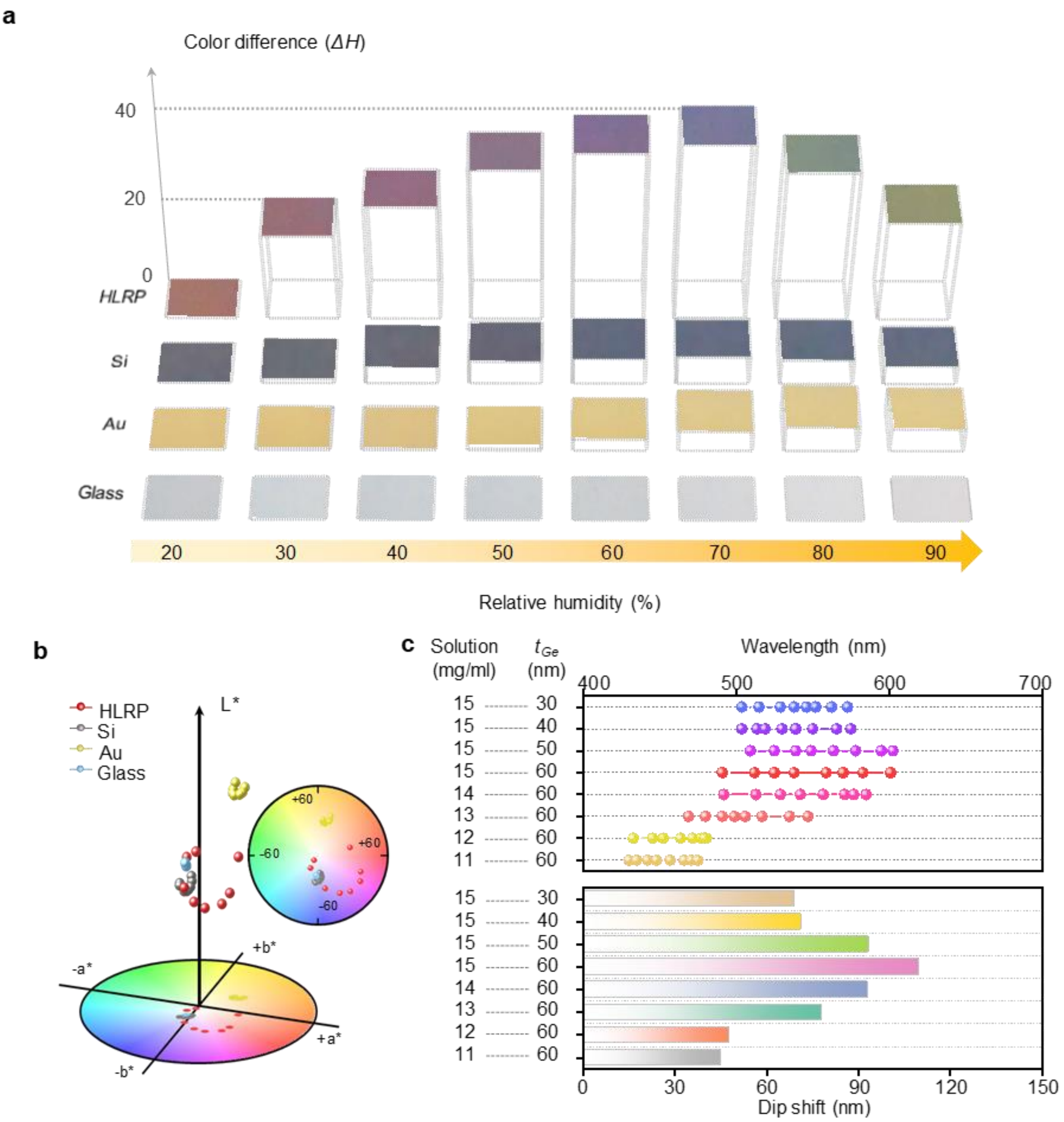

Figure S14. a) Comparison of color differences (*ΔE*) for the phage coated HLRP, Si, Au, and glass. b) 3D chromaticity plot of the optimized phage-based HLRP and phage coated Si, Au, and glass in the CIE L*a*b* space. c) Reflection dip shift with varying phage solution concentrations and HLRP thickness (t_{Ge}).

Supporting Information 14: Insensitive/sensitive color change corresponding to HLRP layer.

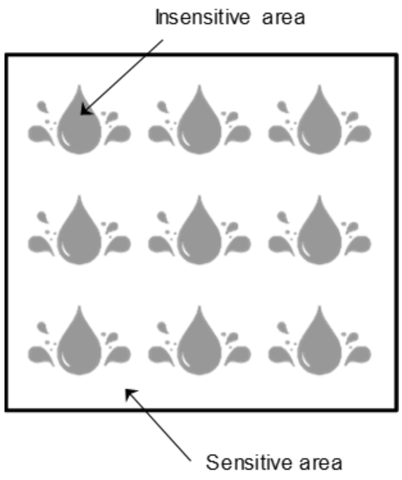

Figure S15. Design of insensitive/sensitive areas of colorimetric sensors with droplet symbols.

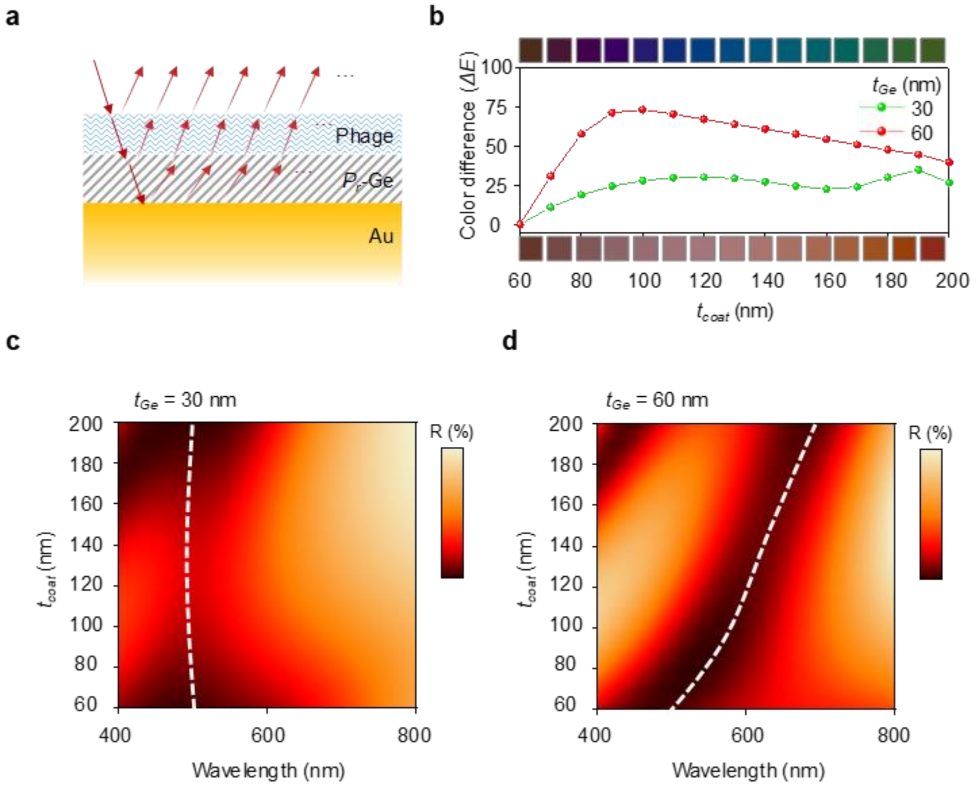

Figure S16. a) Schematic of the phage coated HLRP. b) Color difference (*ΔE*) and color representation with coating thickness (t_{coat}) change. c–d) Reflectance contour as a function of coating thickness (t_{caot} = 60–200 nm) with different HLRPs designed as c) insensitive (t_{Ge} = 30 nm) and d) sensitive (t_{Ge} = 30 nm) areas. White dashed lines present minimum dip shift.

Supporting Information 15: Colorimetric sensor display design and uniformity test.

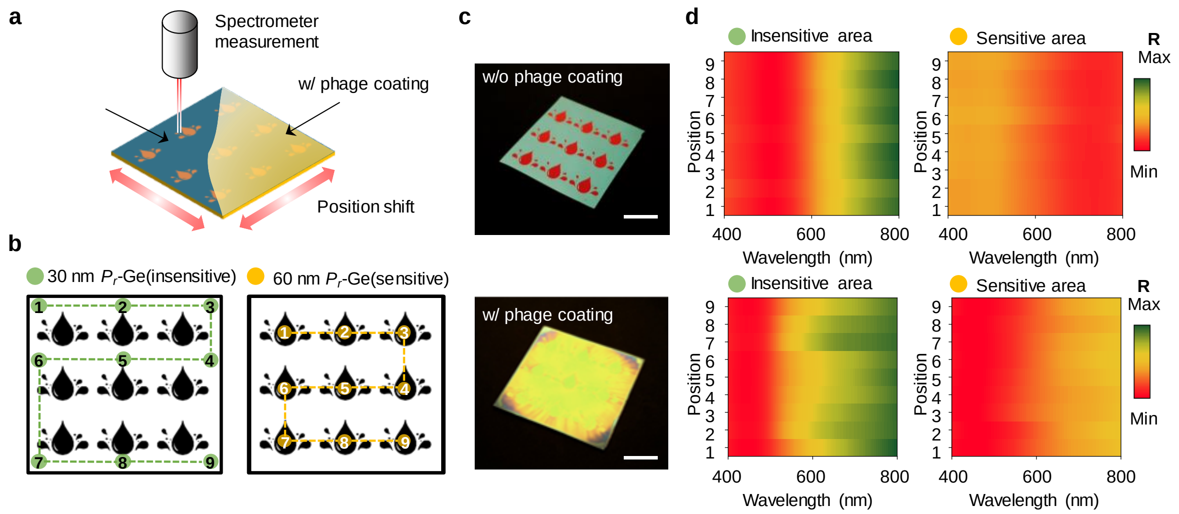

Figure S17. a) Schematic illustration of colorimetric sensor display and measurement setup. b) Measured spot position of insensitive/sensitive region. c) Color images of patterned HLRP (top), and phage-coated sample (bottom). Scale bar is 1 cm. d) Reflectance contours resulting from b). From the dip position, surface uniformity was confirmed over whole area.

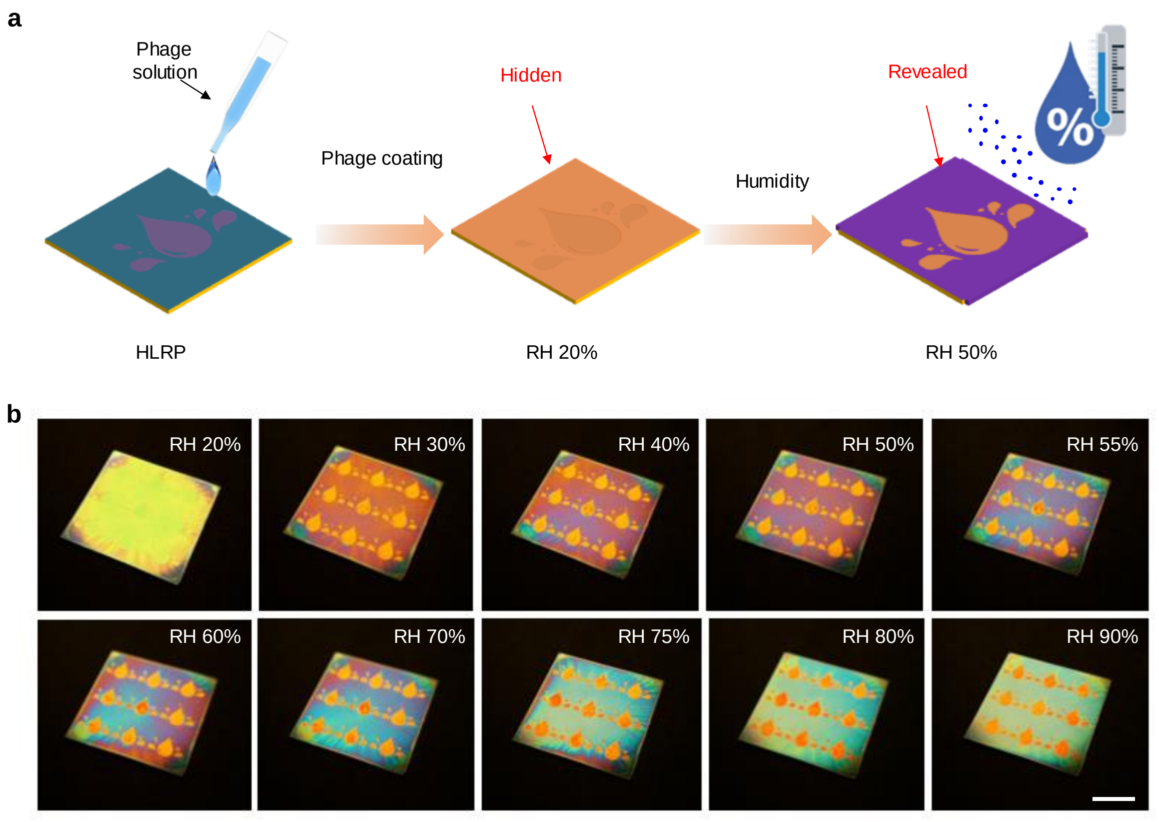

Figure S18. a) Schematic images of colorimetric sensor display for patterned HLRP and phage coated HLRP with humidity change. b) Color images of colorimetric sensor display with humidity change. Scale bar is 1 cm.

Supporting Information 16: Angle dependent property.

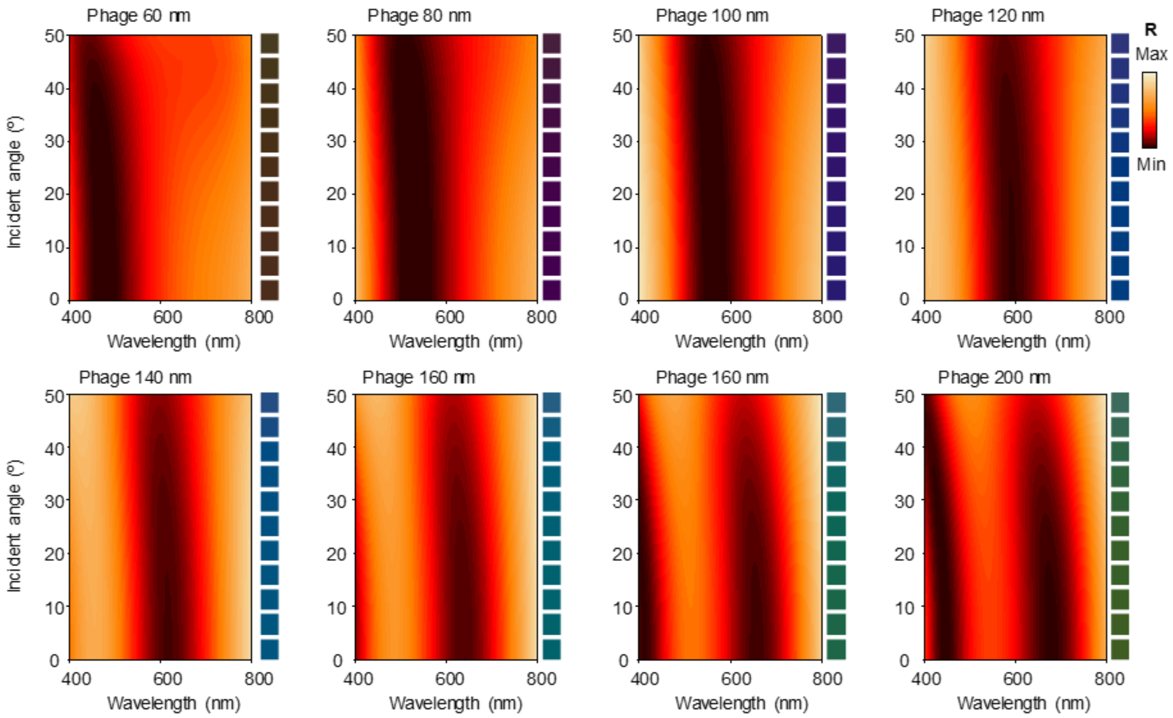

Figure S19. Calculated reflectance of phage coated HLRP with different incident angles in each phage layer thickness (60–200 nm).

Supporting Information 17: Colorimetric indicator display movie.

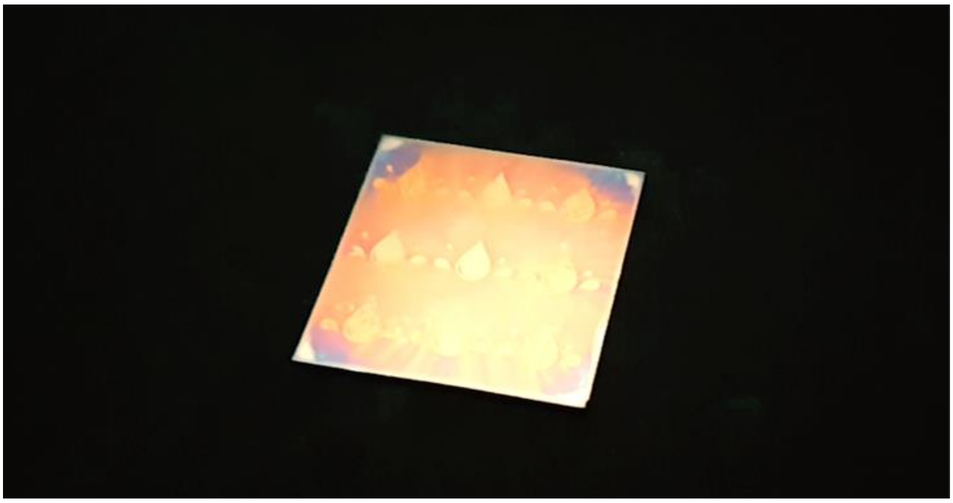

Movie S1. Movie clip showing humidity colorimetric sensor display with breathing.

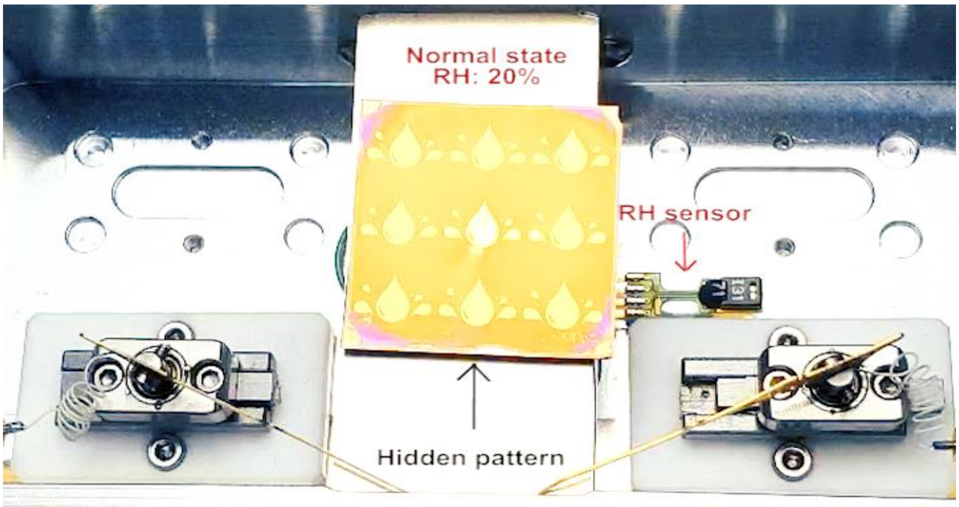

Movie S2. Movie clip showing humidity colorimetric sensor display with moisture flow.

Supporting Information 18: MCSA measurement setup.

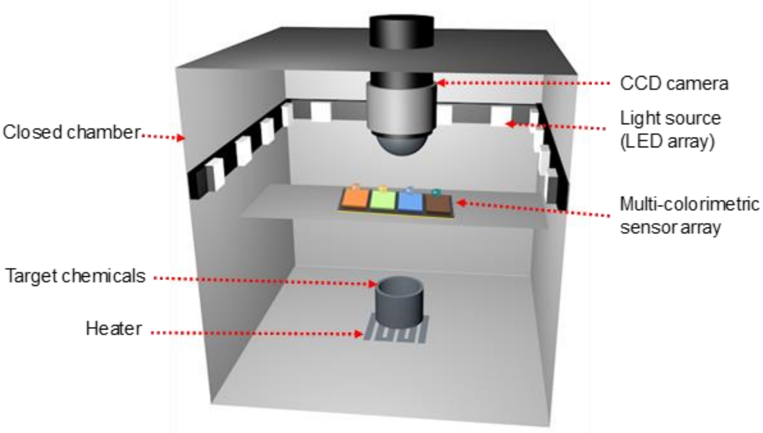

Figure S20. Schematic illustration of experimental setup for volatile organic chemicals (VOCs) and endocrine disrupting chemicals (EDCs) sensing.

Supporting Information 19: Selective response of genetically engineered phage.

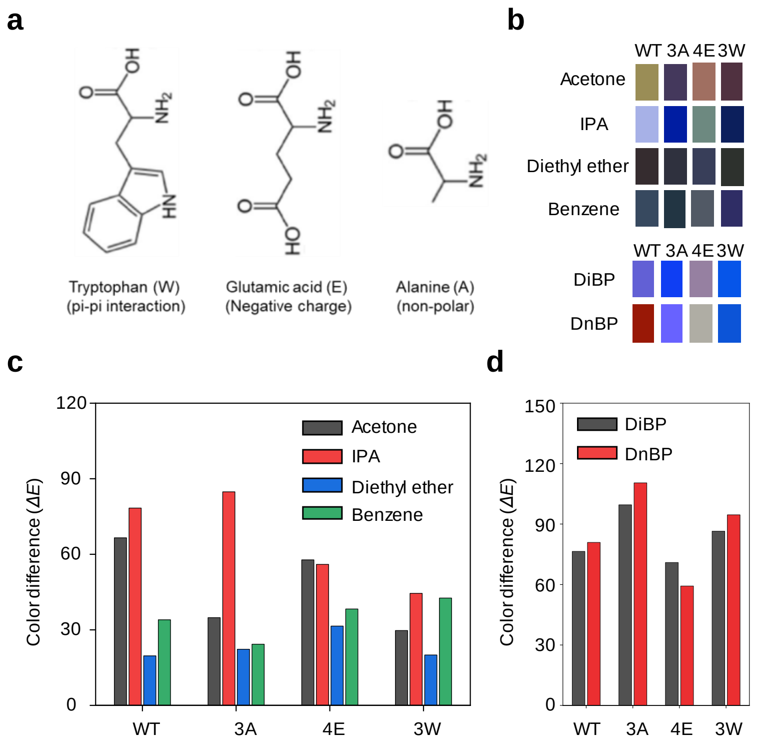

Figure S21. a) Tryptophan(W), glutamic acid(E) and alanine(A) on the phage surface as a receptor for pi-pi stacking interaction, charge interaction and hydrophobic interaction, respectively. b) Color palette of control experiment with various VOCs and EDCs. c-d) Control experiment result between various c) VOCs and d) EDCs with regard to color difference (*ΔE*). As an example in the results, the phage film based upon the 3W-type shows dominant color change towards benzene because of pi-pi interaction between benzene and imidazole of W. In addition to the 3W-type phage, the 4E and wild-type phage showed some color changes and their origin can be probably from electrostatic interaction with a benzene molecule. Because the 4E-type phage has a twice as much charge as the wild-type phage, the 4E-type phage exhibited more color change compared with the wild-type phage. The 3A-type phage also displayed small color change because of a hydrophobic interaction. In this way, multi-array sensor provides fingerprint of exposed molecule which is determined by a difference in binding affinity.

Supporting Information 20: Color information of genetically engineered colorimetric sensor array.

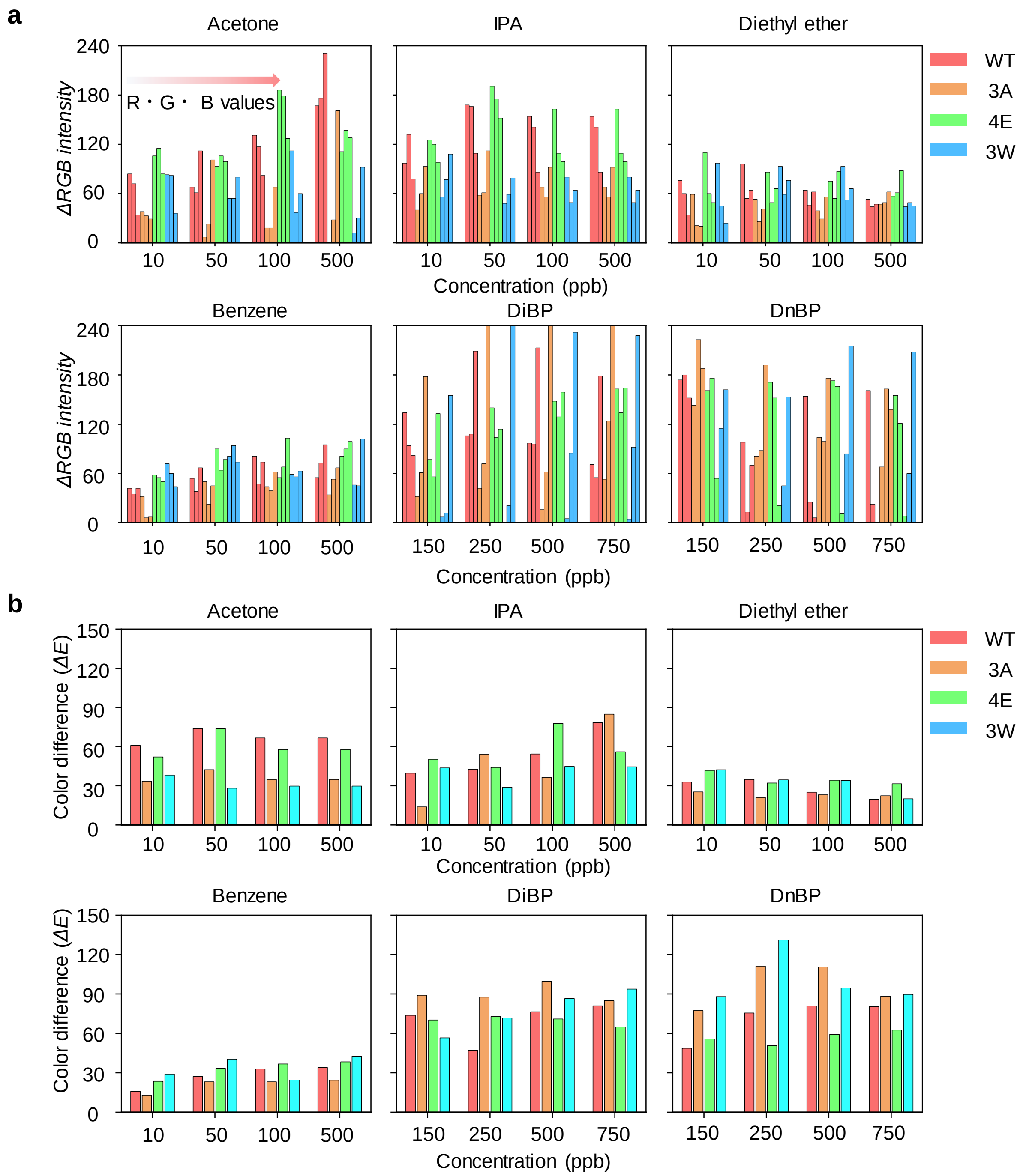

Figure S22. a) *ΔRGB intensity* values of each parts from genetically engineered MCSA corresponding gas concentration change. b) Color differences of each parts from *ΔRGB intensity*.

Supporting Information 21: Control experiment of EDCs.

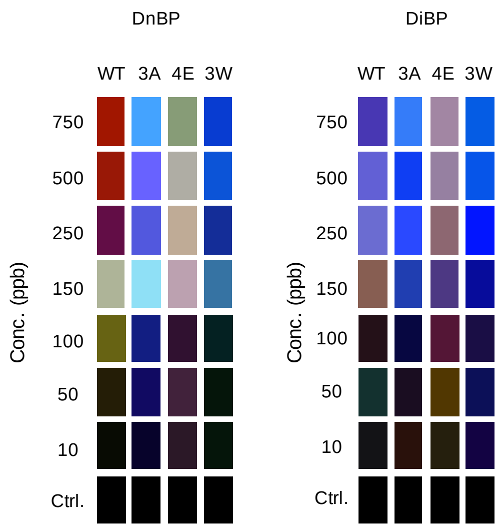

Figure S23. Color palette based on *ΔRGB intensity* with full range concentration change for DnBP and DiBP.

Supporting Information 22: Material information for Permissible exposure limits.

Table S1. Permissible exposure limits of EDCs specified by Occupational Safety and Health Administration (OSHA).[S4]

Molecular formula	Structure	Molecular weight [g/mol]	PEI [mg/m^3]	Concentration [ppb]
Diiscobutyl phthalate (DiBP)	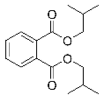	278.35	5	440
Di-n-butyl phthalate (DnBP)	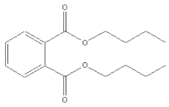	240.21	5	510

Supporting Information 23: Principal component analysis of VOCs and EDCs.

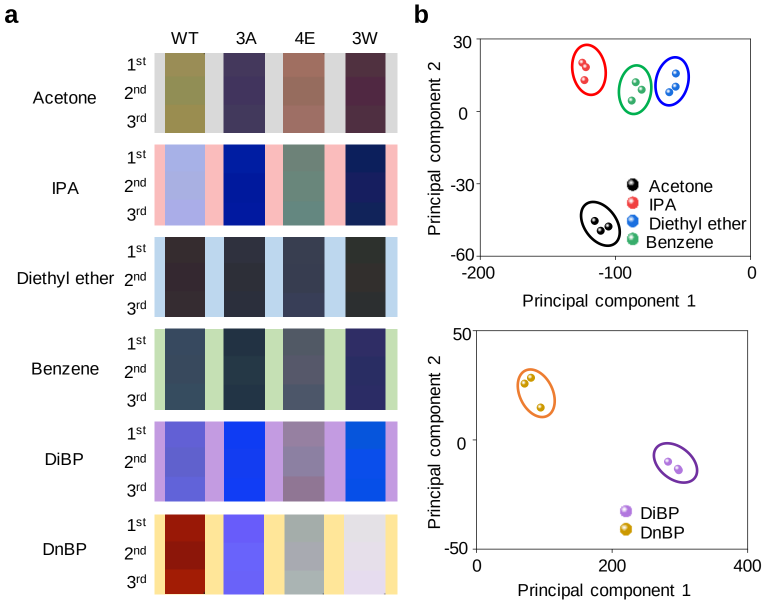

Figure S24. a) Color palette for various VOCs and EDCs. b) Principal component analysis (PCA) results for various VOCs and EDCs. The results show the reliability and reversibility over three times repeated measurement as a colorimetric sensor.

References

[S1] X. Gu, L. Shaw, K. Gu, M. F. Toney, Z. Bao, *Nat. Commun.* **2018**, *9*, 534.

[S2] N. Ohta, A. Robertson, *Colorimetry: Fundamentals and Applications*. John Wiley & Sons, Hoboken, NJ, USA **2006**.

[S3] V. C. Smith, J. Pokorny, *The science of color*. **2003**, *2*, 103.

[S4] OSHA, C*hemical Sampling Information: Phthalate*, https://www.osha.gov/dts/sltc/methods/organic/org104/org104.html (accessed: March 2020).
